# Identification of a gene set that maintains tumorigenicity of the hepatocellular carcinoma cell line Li-7

**DOI:** 10.1007/s13577-023-00967-7

**Published:** 2023-08-23

**Authors:** Yusuke Seyama, Kazuhiro Sudo, Suguru Hirose, Yukako Hamano, Takeshi Yamada, Takashi Hiroyama, Ryosuke Sasaki, Masami Yokota Hirai, Ichinosuke Hyodo, Kiichiro Tsuchiya, Yukio Nakamura

**Affiliations:** 1 Division of Gastroenterology, Faculty of Medicine, University of Tsukuba, Tsukuba, Japan; 2 Cell Engineering Division, RIKEN BioResource Research Center, Tsukuba, Japan; 3 Department of Gastroenterology, Hitachi General Hospital, Hitachi, Japan; 4 Division of Clinical Research and Regional Innovation, Faculty of Medicine, University of Tsukuba, Tsukuba, Japan; 5 RIKEN Center for Sustainable Resource Science, Yokohama, Japan; 6 Department of Gastrointestinal Medical Oncology, National Hospital Organization Shikoku Cancer Center, Matsuyama, Japan

**Keywords:** Cancer stem cell, CD13 expression, Hepatocellular carcinoma, RNA sequencing, Tumorigenicity

## Abstract

The identification and development of therapeutic targets in cancer stem cells that lead to tumor development, recurrence, metastasis, and drug resistance is an important goal in cancer research. The hepatocellular carcinoma cell line Li-7 contains functionally different types of cells. Cells with tumor-forming activity are enriched in cancer stem cell-like CD13^+^CD166^−^ cells and this cell population gradually decreases during culture in conventional culture medium (RPMI1640 containing 10% fetal bovine serum). When Li-7 cells are cultured in mTeSR1, a medium developed for human pluripotent stem cells, CD13^+^CD166^−^ cells, and their tumorigenicity is maintained. Here, we sought to identify the mechanisms of tumorigenicity in this sub-population. We compared gene expression profiles of CD13^+^CD166^−^ cells with other cell sub-populations and identified nine overexpressed genes (*ENPP2*, *SCGN*, *FGFR4*, *MCOLN3*, *KCNJ16*, *SMIM22*, *SMIM24*, *SERPINH1*, and *TMPRSS2*) in CD13^+^CD166^−^ cells. After transfer from mTeSR1 to RPMI1640 containing 10% fetal bovine serum, the expression of these nine genes decreased in Li-7 cells and they lost tumorigenicity. In contrast, when these genes of Li-7 cells were forcibly expressed in cultures using RPMI1640 containing 10% fetal bovine serum, Li-7 cells maintained tumorigenicity. A metabolome analysis using capillary electrophoresis–mass spectrometry showed that two metabolic pathways, “Alanine, aspartate and glutamate metabolism” and “Arginine biosynthesis” were activated in cancer stem-cell-like cells. Our analyses here showed potential therapeutic target genes and metabolites for treatment of cancer stem cells in hepatocellular carcinoma.

**Supplementary Information:**

The online version contains supplementary material available at 10.1007/s13577-023-00967-7.

## Introduction

Hepatocellular carcinoma (HCC) is the fifth most common cancer worldwide and the second leading cause of cancer death in men [1]. Although six systemic therapies have been approved after phase III trials, namely atezolizumab plus bevacizumab, lenvatinib, regorafenib, cabozantinib, ramucirumab, and sorafenib, median overall survival of patients with advanced HCC is still low in the range 10.7–19.2 months [2–7]. Thus, the development of new treatments is desired.

The concept of cancer stem cells (CSCs) has deepened our understanding of the heterogeneity and complexity of cancer [8]. CSCs have the potential for self-renewal and multilineage differentiation and have a central role in tumorigenicity [8]. In general, tumorigenicity depends on reprogramming of cellular metabolism, and glycolysis is predominant metabolic pathway rather than oxidative phosphorylation in cancer cells [9]. This metabolic alteration gains the ability to survive in tumor microenvironments such as hypoxia and low nutrition levels, and is involved in anti-apoptosis [10]. On the other hand, CSCs are dependent on oxidative phosphorylation metabolism, or combined metabolism with high glycolysis, which is important and critical for CSC functions such as stemness, migration, and drug resistance [10]. Since CSCs are resistant to chemotherapy and radiotherapy, they play important roles in tumor development, recurrence, and metastasis [11–15]. Overcoming these roles will require specific treatments that target CSCs.

We previously reported that the human HCC cell line Li-7 contains CSC-like cells, identified as CD13^+^CD166^−^ cells, that have a high potential for tumorigenicity [16]. CD13^+^CD166^−^ cells differentiate into CD13^−^CD166^−^ and CD13^−^CD166^+^ cells during long-term in vitro culture in conventional RPMI 1640 culture medium supplemented with 10% fetal bovine serum (RPMI); this differentiation results in the loss of CSC-like cells and of tumorigenicity. We also reported that CSC-like cells in the Li-7 cell line could be maintained using the mTeSR1 medium developed for pluripotent stem cells. Culture in mTeSR1 significantly increased the tumorigenicity of the CSC-like cells and also influenced CD13/CD166 expressions [17]. Analysis of Li-7 cells cultured in RPMI or mTeSR1 provided an explanation for the reversible expression of CD13 and irreversible expression of CD166. In summary, the Li-7 line possessed three cell populations: (1) CD13^+^CD166^−^ cells with strong tumorigenic activity and CSC characteristics; (2) CD13^−^CD166^−^ cells able to revert to CD13^+^CD166^−^ cells; and (3) CD13^−^CD166^+^ cells that are not tumorigenic and cannot revert to CD166^−^ cells. However, the mechanisms of tumorigenicity and maintenance of CSC-like cells remain unknown. Elucidation of these mechanisms may reveal new therapeutic targets against CSCs in HCC.

In this study, we sought to identify genes that are associated with the tumorigenicity of Li-7 cells by comparing RNA sequences from CSC-like and other cells. Through this comparison, we identified nine candidate genes and subjected these to further analyses. The nine genes functioned to maintain tumorigenicity when they were overexpressed in CD13^+^CD166^−^ cells, but failed to recover it when overexpressed in other cell types. In addition, a metabolome analysis using capillary electrophoresis–mass spectrometry (CE–MS) identified metabolites that might be involved in the tumorigenicity of Li-7 cells.

## Materials and methods

### Cell culture

The human HCC cell line Li-7 (RCB1941) was provided by RIKEN BRC through the National BioResource Project of MEXT, Japan. The cells were cultured in RPMI1640 (Gibco) supplemented with 10% fetal bovine serum (FBS) (SIGMA) or mTeSR1 (STEMCELL Technologies). For culture in mTeSR1, cells were first seeded and cultured overnight in RPMI, washed once with PBS, and then switched to mTeSR1. Cells were incubated at 37 ℃ with 5% partial pressure of CO_2_ in a humidified atmosphere. The medium was changed every 2–3 days until cells were reached at approximately 70 to 80% confluency and one-fourth of cells were reseeded in RPMI as described above.

### Flow cytometric analysis and cell sorting

The following antibodies were used for flow cytometric analysis and cell sorting: allophycocyanin-conjugated anti-human CD13 (BD Biosciences), phycoerythrin-conjugated anti-human CD166 (BD Biosciences), phycoerythrin-Vio770-conjugated anti-human CD166 (Miltenyi Biotech). Cells were stained with fluorescent dye-conjugated antibodies in PBS supplemented with 5% FBS at 4 ℃ for 30 min. The cells were washed once with PBS/5% FBS and re-suspended in 7-AAD (BD Biosciences) in PBS/5% FBS to detect dead cells. Aggregated cells were excluded using an FSC-H/FSC-W dot plot; isotype controls were used to determine negative cell populations. FACS Verse (BD Biosciences) and FACS SORP Aria (BD Biosciences) were used for analysis and cell sorting, respectively.

### RNA sequencing and expression analysis

We collected 1 × 10^6^ cells of three cell populations (CD13^+^CD166^−^ cells, CD13^−^CD166^−^ cells, and CD13^−^CD166^+^ cells) cultured in RPMI and one cell population cultured in mTeSR1 (CD13^+^CD166^−^ cells) by cell sorting. The purity of each population was over 95%. RNA extraction, RNA sequencing, and expression analysis were performed by Hokkaido System Science (Sapporo, Japan). Illumina HiSeq 2500 platform (Illumina) was used for RNA sequencing with a 100-bp read length. The raw sequence data were converted to FastQ format and was mapped to the human genome using TopHat. The data were then normalized and analyzed using Cufflinks.

### Cloning and transduction

Total RNA was extracted from Li-7 cells using TRIzol reagent (Invitrogen) and reverse transcribed to cDNA using SuperScript IV First-Strand Synthesis System (Invitrogen). PCR was performed using Tks Gflex DNA Polymerase (Takara Bio) and extracted genes were directly cloned into an entry vector using a pENTR/D-TOPO Cloning kit (Invitrogen). The cloning primers are listed in Table S1. The entry clones for *SERPINH1* (HGE022338/W01A055O02) and *TMPRSS2* (HGE007275/W01A018D03) were provided by RIKEN BRC through the National BioResource Project of MEXT, Japan [18–21]. Entry clones were converted to lentiviral vectors containing the Tet-On advanced inducible gene expression system by an LR reaction using Gateway LR Clonase II Enzyme mix (Invitrogen). Lentivirus was produced by co-transfection of lentiviral vector and the plasmids pCMV-VSV-G-RSV-Rev (RDB04393) and pCAG-HIF gp (RDB04393), which are required for packaging into 293 T cells by transfection using FuGENE HD transfection reagent (Promega). Candidate genes were introduced into Li-7 cells using lentivirus with auxiliary transfection reagent polybrene (nacalai tesque); the transduced cells were cultured with 10 µg/ml blasticidin (Gibco) to select cells with successful transduction. Doxycycline (1 µg/ml; SIGMA) was added to the culture medium to induce overexpression of the genes. All the procedures were performed according to manufacturer’s instructions where appropriate.

### Quantitative real-time PCR

Cells were collected from culture dishes when reached at 70–80% confluency. Total RNA was extracted from cells using TRIzol reagent and reverse transcribed to cDNA using the SuperScript IV First-Strand Synthesis System. TB Green *Premix Ex Taq* II (Tli RNaseH Plus) (Takara Bio) and a Thermal Cycler Dice Real Time System TP800 (Takara Bio) were used for quantitative real-time PCR according to the manufacturer’s instructions. Expression levels of the genes were calculated using the delta-delta Ct method by comparison with the control housekeeping genes *GAPDH*, *ACTB*, and *B2M*. The primers used are listed in Table S1.

### Tumor formation in mice

Five-week-old female BALB/c *nu*/*nu* nude mice were purchased from CLEA Japan, Inc. (Tokyo, Japan). We made suspensions of 5 × 10^6^ cells in 200 µl of RPMI and injected these subcutaneously into both sides of a recipient mouse. The mice were sacrificed when tumors appeared or at 12 weeks after injection.

### Spheroid formation assay

We seeded 1 × 10^4^ cells into each well of a 96-well NanoCulture plate-MS (ORGANOGENIX) with 100 µl of NanoCulture medium R-type supplemented with 10% FBS-R (ORGANOGENIX). Half of the medium was replaced with new medium twice a week. The number of spheroids with a diameter greater than 100 µm or 200 µm was counted on day 15 using a microscope equipped with a digital camera (DP25, Olympus).

### Chemosensitivity and cell proliferation assay

We performed a chemosensitivity assay by seeding 5 × 10^3^ cells into a 96-well flat bottom plate and incubating the plate overnight at 37 ℃. The medium was replaced with fresh medium containing different concentrations of 5-FU and cell viabilities were measured 72 h later using a Cell Counting Kit-8 (Dojindo). Absorbance at 450 nm was measured using a 2030 Multilabel Reader (ARVO X3; PerkinElmer). Cell viability was assessed by seeding 5 × 10^3^ cells into a 96-well flat bottom plate; a Cell Counting Kit-8 was used to assay cell proliferation at 2, 24, 48, 72, and 96 h of culture in the same manner as the chemosensitivity assay.

### CE–MS analysis

CD166^+^ cells in Li-7 cell cultures were removed using biotin-conjugated anti-human CD166 (Miltenyi Biotech) and Dynabeads M-280 Streptavidin (Invitrogen). Subsequently, 1 × 10^7^ CD166^−^ cells were cultured in mTeSR1 for 1, 4, and 7 weeks and then analyzed. The cultured cells were collected in 2 ml plastic tubes, washed with saline, and pelleted. Metabolome analysis was performed as previously described [22]. Briefly, 1 × 10^7^ cells from each sample were added to 500 µl methanol containing 8 µM of two reference compounds (methionine sulfone for cation and camphor 10-sulfonic acid for anion analysis) and were extracted using a Retsch mixer mill MM310 at a frequency of 27 Hz for 1 min with ɸ5 mm zirconia beads. The extracts were centrifuged at 20,400 × g for 3 min at 4 ℃. Five hundred μl of the methanol solution was then transferred to a new tube. Data were then converted to a csv file and processed using the online software MetaboAnalyst 5.0. After data processing and normalization, correlation analyses, principal component analyses (PCAs), and heatmap clustering analyses were performed. PC1 principal loadings were then calculated for a pathway analysis of the top 30 metabolites.

### Statistics

In Fig. 4B Student’s *t* test was performed to identify statistically significant differences. In Fig. 5 Log-rank test was performed to identify statistically significant differences. A value of *p* < 0.05 was considered significant. Analyses were performed with BellCurve for Excel (Social Survey Research Information Co., Ltd.). Normalization, correlation heatmap analysis, PCA, heatmap clustering analysis, and pathway analysis for CE–MS metabolites were performed using MetaboAnalyst 5.0 (https://www.metaboanalyst.ca). “*Homo sapiens* (KEGG)” library was selected for pathway analysis. FDR < 0.05 and Impact > 0.2 were considered as significant.

## Results

### RNA sequence

**Fig. 1:**
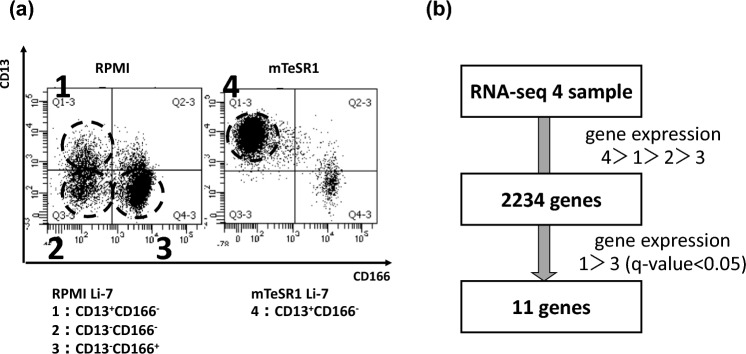
Samples for RNA sequencing and the method for extracting candidate genes. **A** RNA sequencing was performed in three cell fractions that had been cultured in RPMI, namely, CD13^+^CD166^−^ cells (sample 1), CD13^−^CD166^−^ cells (sample 2), and CD13^−^CD166^+^ cells (sample 3), and in one cell fraction that had been cultured in mTeSR1, CD13^+^CD166^−^ cells (sample 4). We collected 1 × 10^6^ cells of each cell fraction by cell sorting. **B** Previous work established that tumorigenicity was in the order sample 4 > sample 1 > sample 2 > sample 3; gene expression levels were extracted in this order. In total, 2234 genes were identified. In addition, the samples with the highest and lowest tumorigenic activity in RPMI, i.e., samples 1 and 3, respectively, were compared and 11 candidate genes with differential gene expression using *q*-value < 0.05 were identified

We performed RNA sequencing to identify genes associated with tumorigenicity in the Li-7 cell line. Three cell populations that had been cultured in RPMI, CD13^+^CD166^−^ cells (sample 1), CD13^−^CD166^−^ cells (sample 2), and CD13^−^CD166^+^ cells (sample 3), and a cell population that had been cultured in mTeSR1, CD13^+^CD166^−^ cells (sample 4), were collected by cell sorting (Fig. 1a). We have previously shown that CD13^+^CD166^−^ cells have tumorigenicity and produce other types of cells such as CD13^−^CD166^−^ and CD13^−^CD166^+^ cells, which have no tumorigenicity activity, in RPMI culture. Cells in both fractions do not show the clear tumorigenic activity, but only CD13^−^CD166^−^ cells can be changed to CD13^+^CD166^−^ cells once culturing in mTeSR1 which is special medium for human iPS cells. Moreover, CD13^+^CD166^−^ cells cultured in mTeSR1 drastically increased tumorigenicity in mice compared to CD13^+^CD166^−^ cells cultured in RPMI. Based on these results, we classified CD13^+^CD166^−^ cells as CSC-like cells, CD13^−^CD166^−^ cells able to change to CSC-like cells, and CD13^−^CD166^+^ cells as terminally differentiated cells and we determined that tumorigenicity from high to low was in the order of samples 4, 1, 2, and 3 [16, 17]. Therefore, we searched for genes that were more abundantly expressed in the order “sample 4 > sample 1 > sample 2 > sample 3”. In other words, the genes with the higher expression levels were sorted in the order of their tumorigenic activity. As a result, 2234 genes were identified (Table S2). Next, we searched for genes showing significantly different expression levels between two cell populations like the following. For sample 4 and sample 1, we considered the possibility that the differences in gene expression levels associated with tumorigenicity were not necessarily large, since both had tumorigenic activity in mice. We selected the genes with significant differences in gene expression levels (*q* value < 0.05) between the cell populations with highest (sample 1) and lowest (sample 3) in RPMI culture. As a result, 11 genes were identified (Fig. 1b, Table 1).

**Table 1:** The 11 candidate genes selected after RNA sequencing

Gene ID	Gene symbol	Official full name	Protein coding
ENSG00000157388	*CACNA1D*	Calcium voltage-gated channel subunit alpha1 D	◯
ENSG00000136960	*ENPP2*	Ectonucleotide Pyrophosphatase/phosphodiesterase 2	◯
ENSG00000079689	*SCGN*	Secretagogin, EF-hand calcium binding protein	◯
ENSG00000160867	*FGFR4*	Fibroblast growth factor receptor 4	◯
ENSG00000055732	*MCOLN3*	Mucolipin 3	◯
ENSG00000153822	*KCNJ16*	Potassium voltage-gated channel subfamily J member 16	◯
ENSG00000267795	*SMIM22*	Small integral membrane protein 22	◯
ENSG00000095932	*SMIM24*	Small integral membrane protein 24	◯
ENSG00000149257	*SERPINH1*	Serpin family H member 1	◯
ENSG00000184012	*TMPRSS2*	Transmembrane serine protease 2	◯
ENSG00000240498	*CDKN2B-AS1*	Cyclin-dependent kinase inhibitor 2B – antisense 1	×

### Overexpression of nine genes in CD13^−^CD166^−^ cells and CD13^−^CD166^+^ cells

**Fig. 2:**
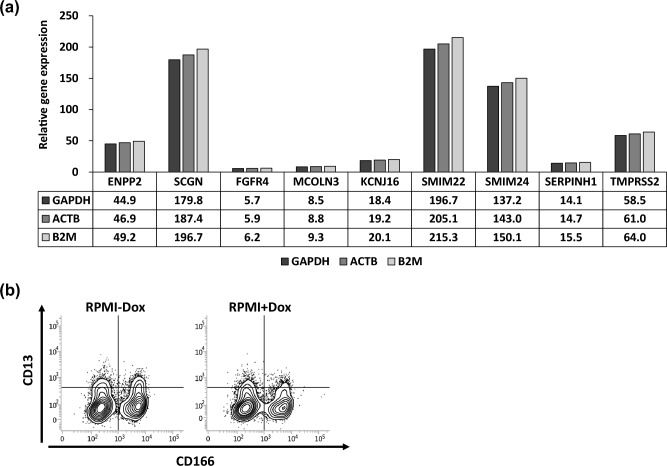
Relative expression levels of transduced genes in 9 g-Li-7 cells. **A** Gene expression levels in 9 g-Li-7 cells were examined by qPCR with the delta-delta Ct method. The expression levels of each gene after the addition of Dox were quantified with respect to the expression levels of each gene without the addition of Dox. *GAPDH*, *ACTB*, and *B2M* were used as housekeeping gene controls, respectively, i.e., the delta-delta Ct method was applied three times separately with each housekeeping gene. **B** Expression profile of CD13 and CD166 on 9 g-Li-7 cells cultured with or without Dox was analyzed with time by flow cytometry

Initially, we tried to overexpress all 11 genes to determine whether they were associated with tumorigenicity. However, two genes, *CACNA1D* and *CDKN2B-AS1,* could not be isolated for some technical reason. The total length of coding sequence of *CACNA1D* was more than 9000 bp for the longest variant, making it difficult to amplify by PCR and unsuitable for expression in the lentiviral vector. As for *CDKN2B-AS1*, a long non-coding RNA with various forms and variants, it was difficult to amplify the poly-A tail without the termination codon by PCR. The remaining nine genes could be isolated and were separately or collectively transduced into Li-7 cells using lentivirus vectors. As is shown in Fig. 2, expression of the transduced genes was inducible by doxycycline (Dox).

Li-7 cells transduced with a single gene and Li-7 cells transduced with all nine genes (9 g-Li-7) were cultured in RPMI with Dox (RPMI + Dox) or without Dox (RPMI-Dox). Overexpression of *ENPP2*, *FGFR4*, and *MCOLN3*, and of all nine genes simultaneously caused delayed cell proliferation but had no significant effect on tumorigenicity in mice, spheroid formation, in chemosensitivity assays, or the expression pattern of CD13 and CD166 (Table S3). The nine genes did not enhance tumorigenicity when overexpressed in Li-7 cells that had been cultured in RPMI over a long period (Table 2a and b). Thus, the nine genes did not recover tumorigenicity in CD13^−^CD166^−^ cells or CD13^−^CD166^+^ cells in the Li-7 line.

**Table 2:** Tumorigenicity of cells transduced with either a single candidate gene or all nine genes (9 g-Li-7)

Gene name	Gene expression	4w	8w	12w
(a)				
* ENPP2*	–	0/2	0/2	0/2
	On	0/2	0/2	0/2
* SCGN*	–	0/2	0/2	0/2
	On	0/2	0/2	0/2
* FGFR4*	–	0/2	0/2	0/2
	On	0/2	0/2	0/2
* MCOLN3*	–	0/2	0/2	0/2
	On	0/2	0/2	0/2
* KCNJ16*	–	0/2	0/2	0/2
	On	0/2	0/2	2/2
* SMIM22*	–	0/2	0/2	0/2
	On	0/2	0/2	0/2
* SMIM24*	–	0/2	0/2	0/2
	On	0/2	0/2	0/2
* SERPINH1*	–	0/2	0/2	0/2
	On	0/2	0/2	0/2
* TMPRSS2*	–	0/2	0/2	0/2
	On	0/2	0/2	0/2
9 genes	–	0/2	0/2	0/2
	On	0/2	0/2	0/2
(b)				
*KCNJ16*	–	0/6	0/6	0/6
	On	0/6	0/6	0/6
9 genes	–	0/4	0/4	0/4
	On	0/4	0/4	0/4

### Overexpression of the nine genes in CSC-like cells (CD13^+^CD166^−^ cells)

**Fig. 3:**
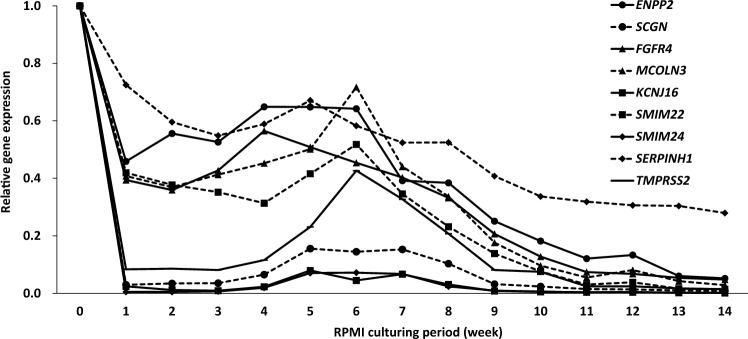
Relative expression levels of nine candidate genes in cells switched from mTeSR1 to RPMI and cultured for different periods. The medium used for culturing CD13^+^CD166^−^ cells was changed from mTeSR1 to RPMI, and expression levels of the nine candidate genes were examined weekly using qPCR

In spite of the results described above, we speculated that the nine genes might still be associated with maintaining tumorigenicity if their expression had decreased with time after changing from mTeSR1 to RPMI (Fig. 3). Switching to RPMI caused the disappearance of CD13^+^CD166^−^ and CD13^−^CD166^−^ cell populations and decreased tumorigenicity in Li-7 cells (Fig. S1a, b). In cells at 16 weeks after changing medium to RPMI, data could not be collected at 8 weeks post-transplant due to a personal technical reason.

**Fig. 4:**
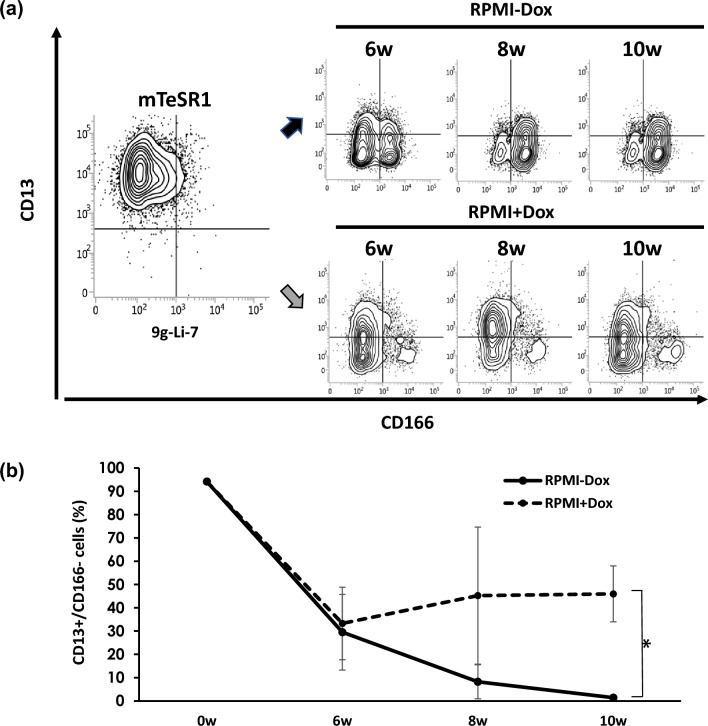
Maintenance of a CSC-like cell population in 9 g-Li-7 cells following overexpression of the transduced nine genes. **A** CD13^+^CD166.^−^ 9 g-Li-7 cells were switched from mTeSR1 to RPMI-Dox (upper columns) or RPMI + Dox (lower columns) medium and cultured for 10 weeks. Expression of CD13 and CD166 was analyzed with time by flow cytometry. **B** Based on flow cytometric analysis, the percentage of CD13-positive cells in RPMI culture with or without Dox was calculated. The data are means ± SD of three separate experiments. *p < 0.05

**Fig. 5:**
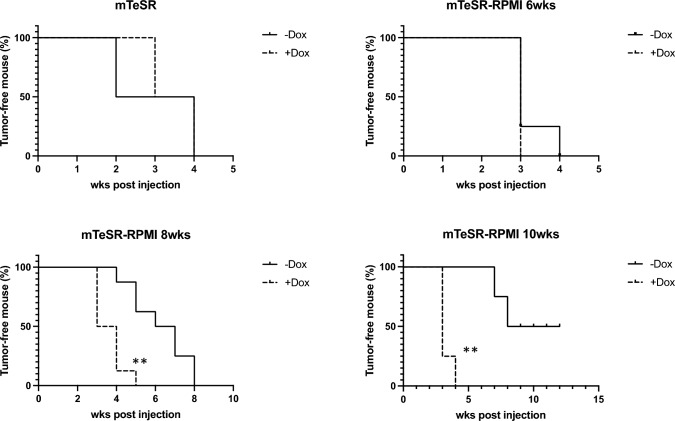
Overexpression of the set of nine genes maintained tumorigenicity in 9 g-Li-7 cells in RPMI culture. CD13^+^CD166^−^ 9 g-Li-7 cells were switched from mTeSR1 to RPMI-Dox or RPMI + Dox and 5 × 10^6^ cells obtained from different time periods of culture were injected subcutaneously to both lateral sides of recipient mice. The proportion of mice with tumor formation at different intervals after injection of CD13^+^CD166.^−^ 9 g-Li-7 cells was assessed. Tumor formation was examined weekly (*n* = 12). **p < 0.01

In the 9 g-Li-7 culture, a significant CD166^−^ population remained after culture with RPMI + Dox compared to RPMI-Dox at 10 weeks after changing the medium to RPMI (Fig. 4A and B). To confirm the roles of the transduced genes in maintaining tumorigenicity, cells were cultured with RPMI + Dox or RPMI-Dox for 6, 8, and 10 weeks and then subcutaneously transplanted into recipient mice. The tumorigenicity of cells cultured with RPMI + Dox was comparable to that of cells cultured with mTeSR1. On the other hand, cells cultured with RPMI-Dox formed fewer tumors and required a longer time period to form tumors (Fig. 5). Other characteristics of CSCs such as high spheroid forming ability, high 5-FU resistance, and slow proliferation were also maintained after 10 weeks of culture with RPMI + Dox (Fig. S2a–d). These results clearly indicate that overexpression of the nine genes resulted in the maintenance of tumorigenicity of Li-7 cells presumably due to the maintenance of a CSC-like cell population.

### CE–MS-based metabolome analysis of Li-7 cells cultured in mTeSR1

**Fig. 6:**
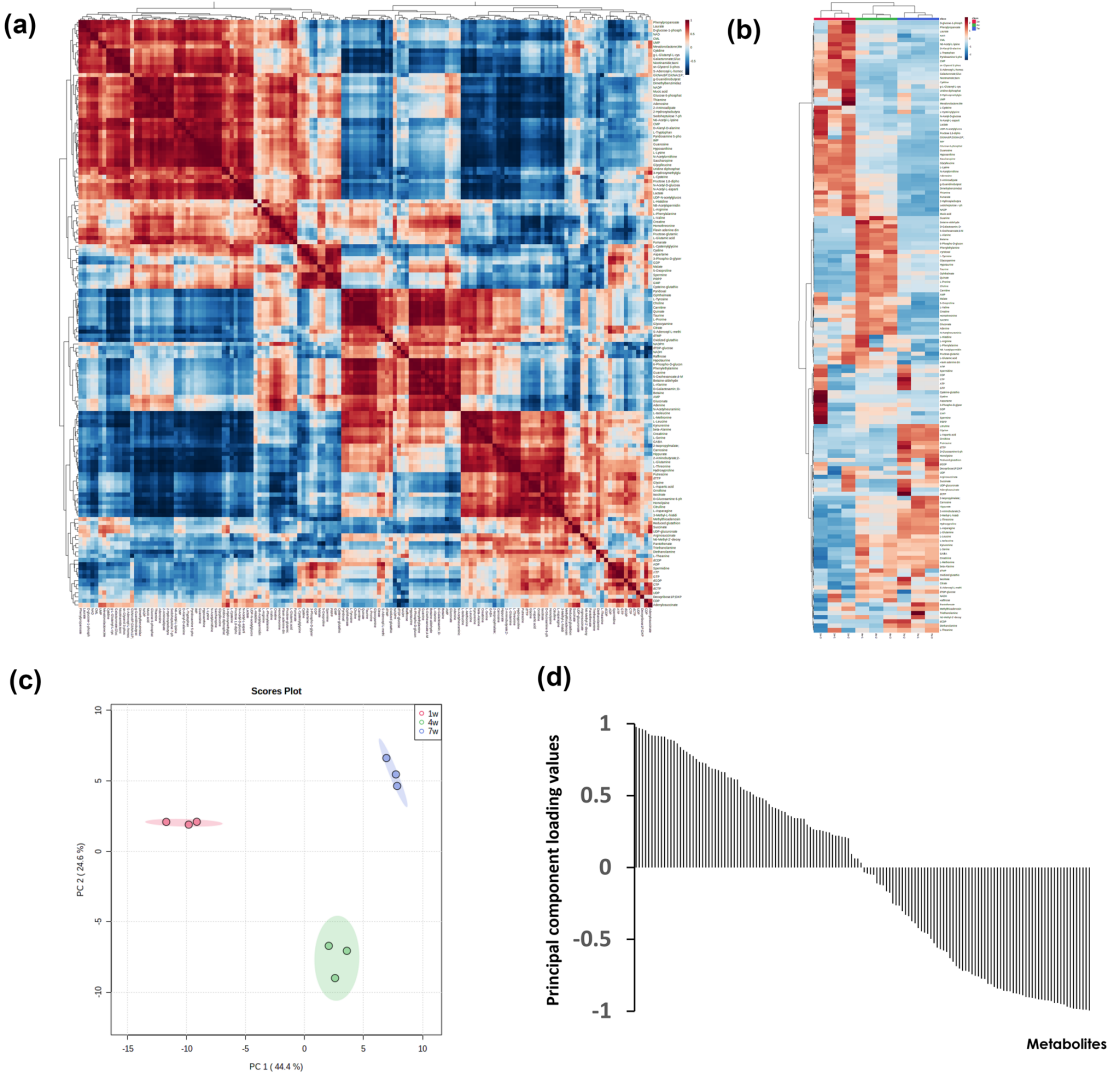
CE–MS analysis. CE–MS analysis detected 144 metabolites that changed in Li-7 cells during culture in mTeSR1 medium. **A** Correlation map and **B** heatmap of metabolites detected by CE–MS. The degree of change is marked with red (upregulation) or blue (downregulation). **C** PCA score plot. Red, 1 week culture in mTeSR1; green, 4 weeks culture in mTeSR1; blue, 7 weeks culture in mTeSR1. **D** Principal component loading of PC1. *ADP* adenosine diphosphate, *AMP* adenosine monophosphate, *ATP* adenosine 5′-triphosphate, *CDP* cytidine diphosphate, *CML* [protein]-N(epsilon)-(carboxymethyl)lysine, *CMP* cytidine monophosphate, *CTP* cytidine 5′-triphosphate, *dCDP* 2′-deoxycytidine 5′-diphosphate, *dGDP* 2′-deoxyguanosine 5′-diphosphate, *dTMP* thymidine 5′-monophosphate, *dTTP* deoxythymidine triphosphate, *GMP* guanosine monophosphate, *GDP* guanosine diphosphate, *GTP* guanosine 5′-triphosphate, *IMP* inosinic acid, *NAD* nicotinamide adenine dinucleotide, *NADH* reduced nicotinamide adenine dinucleotide, *NADP* nicotinamide adenine dinucleotide phosphate, *NADPH* reduced nicotinamide adenine dinucleotide phosphate, *PRPP* phosphoribosyl pyrophosphate, *UDP* uridine 5′-diphosphate, *UMP* uridine monophosphate

The loss of tumorigenicity of Li-7 cells after culture for several weeks in RPMI was largely recovered depending with time after replacement of RPMI with mTeSR1, i.e., the proportion of CD13-positive cells increased immediately after replacing RPMI with mTeSR1 and by Day 7, almost all cells changed to CD13-positive cells (Fig. S3). Recovery of tumorigenicity required culture with mTeSR1 for at least 10 days (Table 3). Metabolome analyses of the CD166^−^ cell population in the Li-7 line after culture for 1, 4, and 7 weeks with mTeSR1 were performed to identify activated metabolic pathways in the CSC-like cells. A total of 144 metabolites were detected in the CE–MS analysis (Fig. 6a). A heatmap clustering analysis and PCA score plots identified differences in metabolites depending on the incubation period with mTeSR1 (Fig. 6b and c). The principal component loadings of PC1 in PCA were calculated (Fig. 6d) and the top 30 metabolites were extracted (Table 4). Pathway analysis of the top 30 metabolites in the PC1 principal loadings, the metabolites enriched in cells cultured with mTeSR1 for 4 or 7 weeks, revealed many pathways showing significant activation (Table 5). Among these pathways, “Alanine, aspartate and glutamate metabolism” and “Arginine biosynthesis” had FDR values < 0.05 and impact values > 0.2.

**Table 3:** Change in tumorigenicity with time after switching cells from RPMI to mTeSR1

Culture condition	1w	2w	3w	4w	5w	6w	7w	8w	9w	10w	11w	12w
RPMI	0/6	0/6	0/6	0/6	0/6	0/6	0/6	0/6	0/6	0/6	1/6	1/6
RPMI → mTeSR1 1 day	0/2	0/2	0/2	0/2	0/2	0/2	0/2	0/2	0/2	0/2	0/2	0/2
RPMI → mTeSR1 3 days	0/2	0/2	0/2	0/2	0/2	0/2	0/2	0/2	0/2	0/2	0/2	0/2
RPMI → mTeSR1 7 days	0/2	0/2	0/2	0/2	0/2	0/2	0/2	0/2	0/2	0/2	0/2	0/2
RPMI → mTeSR1 10 days	0/2	0/2	0/2	0/2	0/2	0/2	0/2	2/2				
RPMI → mTeSR1 14 days	0/2	0/2	0/2	0/2	0/2	0/2	0/2	2/2				
RPMI → mTeSR1 21 days	0/2	2/2										

Recipient mice were injected subcutaneously on both lateral sides with 5 × 10^6^ Li-7 cells cultured for different times in mTeSR1

The mice were screened weekly for tumor formation (*n* = 2 or *n* = 6 were used per treatment group)

**Table 4:** Top 30 metabolites of PC1 principal component loading values

Metabolites	KEGG ID	Principal component loading values of PC1
Hydroxyproline	C01015	0.97737451
L-Glutamine	C00064	0.96889786
L-Threonine	C00188	0.96274028
L-Isoleucine	C00407	0.95298413
L-Asparagine	C00152	0.92819391
Kynurenine	C00328	0.91843776
L-Methionine	C00073	0.91627861
2-Aminobutyrate;2-Aminoisobutyrate	C02261	0.91515905
Hippurate	C01586	0.91132056
Carnosine	C00386	0.90988113
L-Leucine	C00123	0.89164832
3-Methyl-L-histidine	C01152	0.88844959
Creatinine	C00791	0.88109249
beta-Alanine	C00099	0.86245984
L-Serine	C00065	0.83615021
Homolysine		0.81711772
Citrulline	C00327	0.8042428
Isocitrate	C00311	0.78713754
GABA	C00334	0.77563008
D-Glucosamine 6-phosphate	C00352	0.75552601
2-Isopropylmalate;3-Isopropylmalate	C02504, C04411	0.73385456
Oxidized glutathione	C00127	0.7299361
S-Adenosyl-L-methionine	C00019	0.72375454
Ophthalmate	C21016	0.70069164
Diethanolamine	C06772	0.68768877
Pyridoxal	C00250	0.68500982
Putrescine	C00134	0.67714093
Citrate	C00158	0.6647938
Ornithine	C00077	0.66321042
dTTP	C00459	0.62677679

*dTTP* deoxythymidine triphosphate, *PC* principal component

**Table 5:** Pathway analysis of the top 30 metabolites in PC1 principal component loading values

Metabolic pathways	Total	Expected	Hits	Raw p	− log10(*p*)	Holm *p*	FDR	Impact
Aminoacyl-tRNA biosynthesis	48	0.92903	7	2.07E-05	4.6837	0.0017402	0.0017402	0.16667
Alanine, aspartate and glutamate metabolism	28	0.54194	5	0.00013829	3.8592	0.011478	0.0058082	0.20032
Valine, leucine and isoleucine biosynthesis	8	0.15484	3	0.00034365	3.4639	0.02818	0.0096223	0
Arginine biosynthesis	14	0.27097	3	0.0020644	2.6852	0.16722	0.043353	0.28934
Glyoxylate and dicarboxylate metabolism	32	0.61935	4	0.0028159	2.5504	0.22527	0.044258	0.07408
Cysteine and methionine metabolism	33	0.63871	4	0.0031613	2.5001	0.24974	0.044258	0.17901
Arginine and proline metabolism	38	0.73548	4	0.00533	2.2733	0.41574	0.06396	0.24316
Glutathione metabolism	28	0.54194	3	0.01547	1.8105	1	0.16244	0.03417
Histidine metabolism	16	0.30968	2	0.036734	1.4349	1	0.31421	0.09016
Pyrimidine metabolism	39	0.75484	3	0.037406	1.4271	1	0.31421	0

Pathways of metabolites with raw values of *p* < 0.05 are listed in the table

The total number of compounds in the pathways (Total), the expected number of matching compounds in the pathways (Expected), the matched number of compounds (Hits), the original *p* value calculated from the enrichment analysis (Raw *p*), the *p* value adjusted by the Holm–Bonferroni method (Holm *p*), the *p* value adjusted using false discovery rate (FDR), and the pathway impact value calculated from a pathway topology analysis (Impact) of each metabolic pathway are shown

FDR < 0.05 and impact > 0.2 are considered to be significant

## Discussion

CSCs are important targets in treatment strategies for cancers. We previously reported the presence of CSC-like cells in HCC cell line Li-7 with the decrease in number during culture in RPMI, the medium used during generation of Li-7 [16]. In a subsequent study, we searched for a method to maintain CSC-like cells in cultures of Li-7 cells and found that a medium used for human pluripotent stem cell culture, mTeSR1, maintained CSC-like cells and their tumorigenicity [17]. Here, we sought to identify candidate genes associated with tumorigenicity of HCC cells using the Li-7 cell line.

As described here, we extracted genes that are highly expressed in CSC-like cells and overexpression of the nine genes resulted in the maintenance of tumorigenicity of CSC-like cells in Li-7 cell line. One of the nine genes *FGFR4* has been previously implicated in HCC, and the *FGF19*-*FGFR4* pathway in HCC has attracted attention as a potential oncogenic pathway. The combined use of *FGFR4* inhibitors and sorafenib suppresses *FGFR4*/*ERK* signaling and enhances tumor suppression in mice [23]. Moreover, *FGF19*-*FGFR4* signaling facilitates self-renewal of liver CSCs by stimulating the *FGF19*/stimulated store-operated Ca^2+^ entry/nuclear factors of activated T cells (NFAT)-c2 signaling circuit [24]. *FGFR4* may play a pivotal role for tumorigenicity and maintenance of CSC-like cells in the Li-7 line.

Deletion of the gene *ENPP2* results in a reduction of both fibrosis and HCC in mice [25]; *SERPINH1* can function as a biomarker for early-stage HCC [26] and is associated with cell proliferation, migration, and invasion in HCC cell lines [27]. These observations suggest that both of these genes may be related to tumorigenicity of CSC-like cells in the Li-7 line. Although the relevance to HCC is not known, potassium channels, including *KCNJ16*, are involved in cell cycle, proliferation, cell migration, apoptosis, and angiogenesis in various cancers [28, 29]. *TMPRSS2* is also involved in other types of cancer, such as prostate cancer, through its important role in cell invasion, tumor growth, and metastasis [30]. There is, thus, a possibility that *TMPRSS2* is also associated with liver cancer. To the best of our knowledge, there are no reports on the possible involvement of *SCGN*, *MCOLN3*, *SMIM22*, or *SMIM24* with HCC or CSCs; moreover, the functions of *SMIM22* and *SMIM24* have not yet been identified. In a future study, we shall narrow down the identification of essential genes associated with tumorigenesis.

We performed a metabolome analysis here to obtain insights on the tumorigenicity of Li-7 cells. Through this CE–MS analysis, altered metabolites were identified that depended on the culture conditions with mTeSR1. We found that “Alanine, aspartate and glutamate metabolism” and “Arginine biosynthesis” showed significant activation in cells from long-term cultures with mTeSR1 that were tumorigenic. A PCA indicated that the top 30 metabolites in the PC1 loading values associated with these metabolic pathways were L-asparagine, gamma-aminobutyric acid (GABA), L-glutamine, citrate, D-glucosamine 6-phosphate, L-citrulline, and L-ornithine. Of these 7 metabolites, L-asparagine and GABA are frequently reported in relation to tumorigenicity. L-Asparagine is reported as an important regulator of cancer cell proliferation and amino acid homeostasis [31]. Decrease of de novo intracellular asparagine synthesis suppresses tumor growth in a mouse xenograft model [32]. GABA has been reported to be involved in the growth of various cancers including HCC [33]. Decreased expression of gamma-aminobutyrate aminotransferase leads to increased levels of GABA and tumor growth via the Ca^2+^-NFAT1 axis [34]. Furthermore, silencing the GABA-A receptor subunit Pi reduces ERK1/2 phosphorylation and suppresses migration [35]. Thus, L-asparagine and GABA may be involved in the tumorigenicity of Li-7 cells.

Regarding CSC metabolism, metabolic alterations such as in the AKT-mTOR pathway, glutamine metabolism, and fatty acid metabolism regulate self-renewal of stem cells and their function [36]. With regard to lipid metabolism, de novo synthesis of fatty acids from citrates is increased in cancer cells to satisfy energy demands [36]. The expression of enzymes involved in fatty acid de novo synthesis or fatty acids uptake, such as ATP-citrate lyase, acetyl-CoA carboxylase, fatty acid synthase, and fatty acid transporter CD36, is elevated in the CSCs of HCC [36]. These four lipid-related enzymes have been found to be most highly expressed in CD13^+^CD166^−^ cells cultured with mTeSR1 among the four cell samples used for RNA sequencing analysis (data not shown). This information and the finding of increased intracellular citrate concentrations in the CE–MS analysis suggest that lipid metabolism may be involved in the mechanisms maintaining CSC-like cells in the Li-7 line. The results obtained from metabolome analysis may be specific for cell line. However, identifying metabolic pathways that are activated in cells with high tumorigenicity may provide clues to controlling CSCs in vivo. There is a possibility that the metabolic pathways including the metabolites identified from Li-7 are involved in tumorigenicity of HCC. However, further analyses of primary cancer cells are necessary to confirm this hypothesis. Therefore, analyzing primary cancer cells must be an important next step to further elucidate the metabolic pathways involved in tumorigenicity of HCC.

Our study was initiated to identify genes associated with the tumorigenicity of CSC-like cells in the HCC cell line Li-7. As a result, nine genes were found to be associated with maintenance of CSC-like cells and their tumorigenicity. In addition, some metabolites seemed to be associated with tumorigenicity. Although the relationship between the genes and metabolites requires further study, it is interesting to note that there are reports on Ca^2+^/NFAT1 (or NFATc2) axis and ERK signaling for the gene *FGFR4* and the metabolite GABA. In our next study, we shall investigate these nine genes further with the aim of identifying targets for therapy against CSCs in HCC. It is possible that other genes, in addition to the nine described here, are associated with the maintenance of CSCs and tumorigenicity in HCC. The Li-7 cell line will be valuable to identify other such genes in future research.

### Supplementary Information

Below is the link to the electronic supplementary material.

Supplementary file1 (PDF 1585 KB)

Supplementary file2 (PDF 311 KB)
