# Supplementary material for: Identification of a gene set that maintains tumorigenicity of the hepatocellular carcinoma cell line Li-7
Source: Hum Cell. 2023 Aug 23;36(6):2074–86. doi: 10.1007/s13577-023-00967-7 (PMC10587214; doi:10.1007/s13577-023-00967-7)
Supplement: Supplementary file 1 — Supplementary file1 (PDF 1585 KB) [file 13577_2023_967_MOESM1_ESM.pdf]

(a)

Culture condition	1w	2w	3w	4w	5w	6w	7w	8w	9w	10w	11w	12w
mTeSR1	**2/2**											
mTeSR1→RPMI **4w**	0/0	**2/2**										
mTeSR1→RPMI **8w**	0/0	**2/2**										
mTeSR1→RPMI **12w**	0/2	0/2	**1/2**	**2/2**								
mTeSR1→RPMI **16w**	0/2	0/2	0/2	0/2	0/2	0/2	0/2	-	**1/2**	**1/2**	**2/2**	
mTeSR1→RPMI **20w**	0/2	0/2	0/2	0/2	0/2	0/2	0/2	0/2	0/2	0/2	**1/2**	**2/2**

(b)

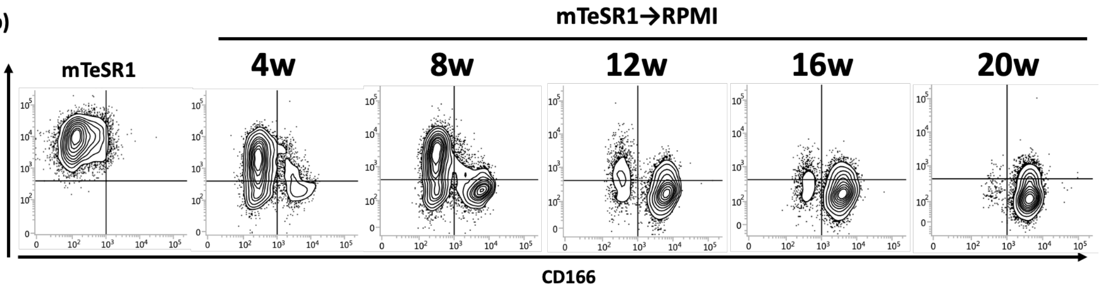

Fig. S1: Changes in tumorigenicity and CD13/CD166 phenotype after transferring cells from mTeSR1 to RPMI.

(A) Tumorigenicity of 5 x 10^6 cells injected subcutaneously to both lateral sides of a recipient mouse. The cells were cultured for 0, 4, 8, 12, 16, or 20 weeks after transfer from mTeSR1 to RPMI. For a personal technical reason, the data from 8w after transferring medium to mTeSR1 was not obtained. Tumor formation was examined weekly (n = 2).

(B) CD13 and CD166 expression was analyzed by flow cytometry with time after transfer from mTeSR1 to RPMI.

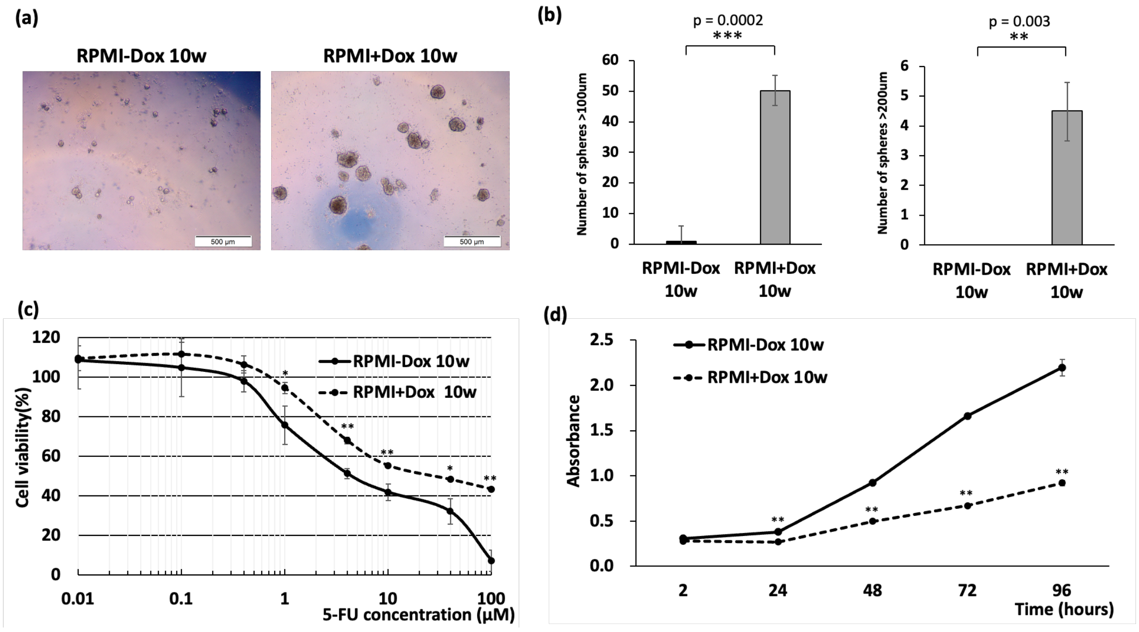

Fig. S2: Overexpression of the transduced nine genes supports maintenance of the CSC characteristics of 9g-Li-7 in RPMI culture.

9g-Li-7 cells were cultured for 10 weeks after switching to RPMI with or without Doxycycline.

(A) and (B) spheroid forming assay. 1 x 10^4 cells were seeded into a 96-well NanoCulture plate-MS and the numbers of spheroids with a diameter greater than 100 µm or 200 µm were counted on day 15 using a microscope. (A) Spheroid morphology. Magnification, x40. (B) Number of spheroids over 100 µm and 200 µm. Values are means ± SD of 4 wells (**p < 0.01, ***p < 0.001).

(C) Chemosensitivity assay. 5 x 10^3 cells were seeded into a 96-well flat bottom plate and incubated at 37°C overnight. Medium was replaced with fresh medium containing different concentrations of 5-FU; cell viabilities were measured as absorbance 72 hours later using Cell Counting Kit-8. Values are means ± SD of 3 wells (*p < 0.05, **p < 0.01).

(D) Proliferation assay. 5 x 10^3 cells were seeded into a 96-well flat bottom plate and cell viabilities were measured as absorbance 2, 24, 48, 72, and 96 hours later using Cell Counting Kit-8. Values are means ± SD of 3 wells (**p < 0.01).

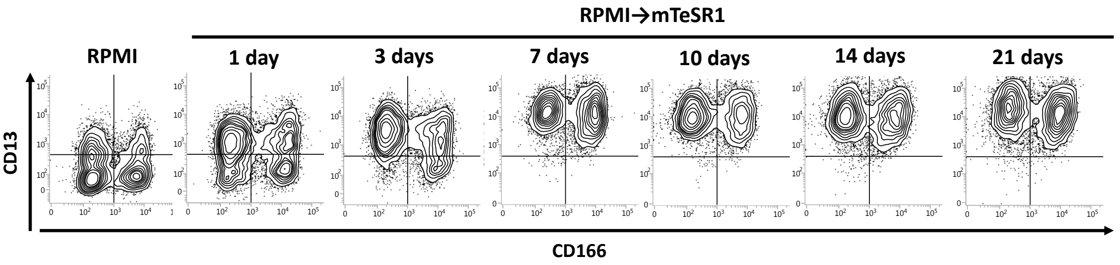

Fig. S3: Changes in CD13 and CD166 expression profiles over time after changing media to mTeSR1. Li-7 cells cultured in RPMI were switched to mTeSR1 and expression of CD13 and CD166 was analyzed with time by flow cytometry.
