# Supplementary material for: Identification of a gene set that maintains tumorigenicity of the hepatocellular carcinoma cell line Li-7
Source: Hum Cell. 2023 Aug 23;36(6):2074–86. doi: 10.1007/s13577-023-00967-7 (PMC10587214; doi:10.1007/s13577-023-00967-7)
Supplement: Supplementary file 2 — Supplementary file2 (PDF 311 KB) [file 13577_2023_967_MOESM2_ESM.pdf]

Table S1: The List of primers

Gene name	Cloning primers	Quantitive real-time PCR primers
GAPDH		5’-CGCTCTCTGCTCCTCCTGTT-3’<br>5’-CCATGGTGTCTGAGCGATGT-3’
ACTB		5’-CTGGAACGGTGAAGGTGACA-3’<br>5’-AAGGGACTTCCTGTAACAACGCA-3’
B2M		5’-AGGACTGGTCTTTCTATCTCTTG-3’<br>5’-CGGCATCTTCAAACCTCCAT-3’
ENPP2	5’-CACCTCCAAGAATCCTCGACATGGC-3’<br>5’-CAGATGCTCAGAAAGTTAAATCTC-3’	5’-ACCTTCCCAAACGTTTGCAC-3’<br>5’-AGGTTTCCTTGCAACATGCC-3’
SCGN	5’- CACCATACGGTGAAGGAGTCCTTC-3’<br>5’-AGCAGTCTGGGATTATGGGTTGAT-3’	5’-TGCACAAGGTGAAACAGCAG-3’<br>5’-AGCTCTTTCATCCGAATGCG-3’
FGFR4	5’- CACCGAGCTGTGAGAAGGAGATGCGGCTGC-3’<br>5’-CCTGCACAGCCTTGAGCCTTGCTCATG-3’	5’-TAGTACGTGCAGAGGCCTTTG-3’<br>5’-AGGCGTTGTCTTTGAGCATC-3’
MCOLN3	5’-CACCGGCTGCTGGAGTCGCTCGCTGACT-3’<br>5’-GTACAGATAAACCTGATAGCTACT-3’	5’-TGGCAATCTGTCAGCACTTC-3’<br>5’-TTTCTGCTGGTGTCCCAATG-3’
KCNJ16	5’-CACCATGAGCTATTACGGCAGCAGCTATCAT-3’<br>5’-TCATAATTGCAATTTAGGACTACAT-3’	5’-TTGCAGTGCCAAGCAATTGG-3’<br>5’-TTCACAGCTGCTGACAATGG-3’
SMIM22	5’- CACCGCCAAGATGGCTGTGTCCACAGAGG-3’<br>5’-AAGGCTTTGTTACTGCCACCG-3’	5’-GAAGAGCCACCAGTTTTTCCAG-3’<br>5’-GCCCATGAAGGTGAGGAAAAC-3’
SMIM24	5’- CACCGTCATGGAGACCCTGGGGGCCCTT-3’<br>5’-GCAGCCAGGAATCTTCACATGACTGT-3’	5’-AAGGAAGGAGAGAGCAACTTGG-3’<br>5’-CACATGACTGTGCTCTTTGCTC-3’
SERPINH1		5’-ATGCAGAAGAAGGCTGTTGC-3’<br>5’-AAGTCGGCCTTGTTCTTGTC-3’
TMPRSS2		5’-ACAGTGTGCACCTCAAAGAC-3’<br>5’-ACTTGCTGCCCATGAACTTC-3’

Table S2: List of 2234 genes abundantly expressed in the order “sample 4 > sample 1 > sample 2 > sample 3”

Gene ID	Gene short names
ENSG00000273259	AL049839.2
ENSG00000259053	AL137230.2
ENSG00000283239	KBTBD11-OT1
ENSG00000272897	AL109827.1
ENSG00000269242	AC010422.3
ENSG00000283496	AL360181.5
ENSG00000269026	AC003006.1
ENSG00000108242	CYP2C18
ENSG00000272514	CFAP206
ENSG00000228697	AL023755.1
ENSG00000167774	AC010323.1
ENSG00000229186	ADAM1A
ENSG00000172296	SPTLC3
ENSG00000175311	ANKS4B
ENSG00000165186	PTCHD1
ENSG00000130234	ACE2
ENSG00000105852	PON3
ENSG00000147003	TMEM27
ENSG00000167772	ANGPTL4
ENSG00000272679	AL355987.2
ENSG00000276644	DACH1
ENSG00000138823	MTTP
ENSG00000141744	PNMT
ENSG00000173114	LRRN3
ENSG00000109854	HTATIP2
ENSG00000002933	TMEM176A
ENSG00000006042	TMEM98
ENSG00000260122	AC068987.2
ENSG00000198758	EPS8L3
ENSG00000106565	TMEM176B
ENSG00000179913	B3GNT3
ENSG00000163624	CDS1
ENSG00000104537	ANXA13
ENSG00000240498	CDKN2B-AS1
ENSG00000060982	BCAT1
ENSG00000139209	SLC38A4
ENSG00000075388	FGF4
ENSG00000245017	LINC02453
ENSG00000127074	RGS13
ENSG00000099284	H2AFY2
ENSG00000055732	MCOLN3
ENSG00000088386	SLC15A1
ENSG00000242288	AC022400.3

ENSG00000076770	MBNL3
ENSG00000139515	PDX1
ENSG00000187210	GCNT1
ENSG00000248871	TNFSF12-TNFSF13
ENSG00000101076	HNF4A
ENSG00000116771	AGMAT
ENSG00000149573	MPZL2
ENSG00000213214	ARHGEF35
ENSG00000174358	SLC6A19
ENSG00000162882	HAAO
ENSG00000161638	ITGA5
ENSG00000188643	S100A16
ENSG00000134240	HMGCS2
ENSG00000267188	AC245748.2
ENSG00000128298	BAIAP2L2
ENSG00000151572	ANO4
ENSG00000169862	CTNND2
ENSG00000222001	AC106876.1
ENSG00000118271	TTR
ENSG00000187955	COL14A1
ENSG00000138109	CYP2C9
ENSG00000138207	RBP4
ENSG00000277669	AC009086.2
ENSG00000039068	CDH1
ENSG00000134709	HOOK1
ENSG00000050327	ARHGEF5
ENSG00000134548	SPX
ENSG00000110693	SOX6
ENSG00000136881	BAAT
ENSG00000157388	CACNA1D
ENSG00000128268	MGAT3
ENSG00000168811	IL12A
ENSG00000184956	MUC6
ENSG00000258793	AL355102.4
ENSG00000166126	AMN
ENSG00000101349	PAK5
ENSG00000156103	MMP16
ENSG00000112494	UNC93A
ENSG00000127831	VIL1
ENSG00000160588	MPZL3
ENSG00000055957	ITIH1
ENSG00000107736	CDH23
ENSG00000135220	UGT2A3
ENSG00000162738	VANGL2
ENSG00000171747	LGALS4

ENSG00000182795	C1orf116
ENSG00000166394	CYB5R2
ENSG00000250889	LINC01336
ENSG00000131477	RAMP2
ENSG00000065361	ERBB3
ENSG00000186212	SOWAHB
ENSG00000162881	OXER1
ENSG00000282221	AC119427.1
ENSG00000160862	AZGP1
ENSG00000134824	FADS2
ENSG00000005421	PON1
ENSG00000120162	MOB3B
ENSG00000255176	AP000941.1
ENSG00000116014	KISS1R
ENSG00000205744	DENND1C
ENSG00000204219	TCEA3
ENSG00000176153	GPX2
ENSG00000130988	RGN
ENSG00000099834	CDHR5
ENSG00000196876	SCN8A
ENSG00000234155	LINC02535
ENSG00000260372	AQP4-AS1
ENSG00000095585	BLNK
ENSG00000186148	AC013268.1
ENSG00000185565	LSAMP
ENSG00000164764	SBSPON
ENSG00000169856	ONECUT1
ENSG00000156453	PCDH1
ENSG00000163485	ADORA1
ENSG00000180745	CLRN3
ENSG00000165617	DACT1
ENSG00000213759	UGT2B11
ENSG00000145642	FAM159B
ENSG00000170608	FOXA3
ENSG00000111339	ART4
ENSG00000169836	TACR3
ENSG00000137561	TTPA
ENSG00000198053	SIRPA
ENSG00000262528	AL022341.2
ENSG00000234502	FYTTD1P1
ENSG00000234373	SNX18P7
ENSG00000236924	AL162411.1
ENSG00000135917	SLC19A3
ENSG00000173546	CSPG4
ENSG00000110400	NECTIN1

ENSG00000163412	EIF4E3
ENSG00000115423	DNAH6
ENSG00000185442	FAM174B
ENSG00000165841	CYP2C19
ENSG00000111783	RFX4
ENSG00000149257	SERPINH1
ENSG00000152284	TCF7L1
ENSG00000249853	HS3ST5
ENSG00000242193	AL359075.1
ENSG00000108950	FAM20A
ENSG00000250799	PRODH2
ENSG00000169213	RAB3B
ENSG00000188582	PAQR9
ENSG00000274979	AC020656.2
ENSG00000197249	SERPINA1
ENSG00000114378	HYAL1
ENSG00000145321	GC
ENSG00000105707	HPN
ENSG00000138613	APH1B
ENSG00000163631	ALB
ENSG00000164669	INTS4P1
ENSG00000204396	VWA7
ENSG00000197291	RAMP2-AS1
ENSG00000224122	POU6F2-AS1
ENSG00000236383	LINC00854
ENSG00000245156	AP001107.1
ENSG00000163581	SLC2A2
ENSG00000165272	AQP3
ENSG00000169783	LINGO1
ENSG00000120915	EPHX2
ENSG00000230596	GPAA1P2
ENSG00000154274	C4orf19
ENSG00000187688	TRPV2
ENSG00000219607	PPP1R3G
ENSG00000172995	ARPP21
ENSG00000077238	IL4R
ENSG00000264304	AC024267.3
ENSG00000232284	GNG12-AS1
ENSG00000160867	FGFR4
ENSG00000137825	ITPKA
ENSG00000135127	BICDL1
ENSG00000079308	TNS1
ENSG00000137285	TUBB2B
ENSG00000154548	SRSF12
ENSG00000164175	SLC45A2

ENSG00000128917	DLL4
ENSG00000115226	FNDC4
ENSG00000157087	ATP2B2
ENSG00000184160	ADRA2C
ENSG00000167701	GPT
ENSG00000170271	FAXDC2
ENSG00000019144	PHLDB1
ENSG00000122591	FAM126A
ENSG00000120057	SFRP5
ENSG00000125895	TMEM74B
ENSG00000143554	SLC27A3
ENSG00000180190	TDRP
ENSG00000106078	COBL
ENSG00000278621	AC037198.3
ENSG00000111679	PTPN6
ENSG00000049540	ELN
ENSG00000276542	AC097478.3
ENSG00000269481	AC010319.4
ENSG00000211592	IGKC
ENSG00000255524	NPIPB8
ENSG00000139998	RAB15
ENSG00000168077	SCARA3
ENSG00000170439	METTL7B
ENSG00000229851	ARSD-AS1
ENSG00000185499	MUC1
ENSG00000273338	AC103591.3
ENSG00000068078	FGFR3
ENSG00000183145	RIPPLY3
ENSG00000149218	ENDOD1
ENSG00000257150	PGAM1P5
ENSG00000171766	GATM
ENSG00000163406	SLC15A2
ENSG00000108551	RASD1
ENSG00000269416	LINC01224
ENSG00000114638	UPK1B
ENSG00000128242	GAL3ST1
ENSG00000283597	FAM169B
ENSG00000068976	PYGM
ENSG00000166323	C11orf65
ENSG00000183578	TNFAIP8L3
ENSG00000162817	C1orf115
ENSG00000006756	ARSD
ENSG00000281657	LINC00976
ENSG00000137960	GIPC2
ENSG00000166825	ANPEP

ENSG00000153982	GDPD1
ENSG00000169429	CXCL8
ENSG00000260704	LINC00543
ENSG00000061455	PRDM6
ENSG00000179104	TMTC2
ENSG00000104419	NDRG1
ENSG00000182327	GLTPD2
ENSG00000152931	PART1
ENSG00000228748	AL450306.1
ENSG00000116667	C1orf21
ENSG00000166589	CDH16
ENSG00000275202	AL161421.1
ENSG00000117834	SLC5A9
ENSG00000120318	ARAP3
ENSG00000162415	ZSWIM5
ENSG00000139219	COL2A1
ENSG00000235872	AC078777.1
ENSG00000248079	DPH6-AS1
ENSG00000160188	RSPH1
ENSG00000179761	PIPOX
ENSG00000235903	CPB2-AS1
ENSG00000118094	TREH
ENSG00000148584	A1CF
ENSG00000116017	ARID3A
ENSG00000016391	CHDH
ENSG00000266094	RASSF5
ENSG00000160145	KALRN
ENSG00000179588	ZFPM1
ENSG00000277151	AL138820.1
ENSG00000076716	GPC4
ENSG00000198650	TAT
ENSG00000251023	AC114980.1
ENSG00000187867	PALM3
ENSG00000139988	RDH12
ENSG00000223841	AC006484.1
ENSG00000258951	KRT18P7
ENSG00000264127	SCML2P1
ENSG00000255468	AP001107.9
ENSG00000154639	CXADR
ENSG00000198277	AC021146.1
ENSG00000125170	DOK4
ENSG00000198237	AC131392.1
ENSG00000271366	AC002128.2
ENSG00000262362	AC004233.1
ENSG00000256564	AC007552.2

ENSG00000171885	AQP4
ENSG00000248763	AC111000.2
ENSG00000255145	STX17-AS1
ENSG00000227220	AL133346.1
ENSG00000225762	LINC01389
ENSG00000228222	AC073050.1
ENSG00000146250	PRSS35
ENSG00000005471	ABCB4
ENSG00000115604	IL18R1
ENSG00000042062	RIPOR3
ENSG00000274475	RN7SL650P
ENSG00000151014	NOCT
ENSG00000273045	C2orf15
ENSG00000267615	AC087289.4
ENSG00000179361	ARID3B
ENSG00000144891	AGTR1
ENSG00000226562	CYP4F26P
ENSG00000109193	SULT1E1
ENSG00000256966	AL513165.2
ENSG00000258311	AC009779.3
ENSG00000066248	NGEF
ENSG00000176928	GCNT4
ENSG00000137801	THBS1
ENSG00000141519	CCDC40
ENSG00000272983	AL117339.4
ENSG00000172716	SLFN11
ENSG00000221994	ZNF630
ENSG00000259075	POC1B-GALNT4
ENSG00000132437	DDC
ENSG00000152926	ZNF117
ENSG00000251138	AC090502.1
ENSG00000005379	TSPOAP1
ENSG00000174669	SLC29A2
ENSG00000068615	REEP1
ENSG00000155666	KDM8
ENSG00000205795	CYS1
ENSG00000106025	TSPAN12
ENSG00000140057	AK7
ENSG00000169507	SLC38A11
ENSG00000263847	AP005899.1
ENSG00000166823	MESP1
ENSG00000279133	AC018628.1
ENSG00000248740	LINC02428
ENSG00000005187	ACSM3
ENSG00000280138	AC027290.2

ENSG00000179094	PER1
ENSG00000183783	KCTD8
ENSG00000079691	CARMIL1
ENSG00000259953	AL138756.1
ENSG00000231772	Z93403.1
ENSG00000136100	VPS36
ENSG00000234449	FAM239A
ENSG00000262772	LINC01977
ENSG00000225889	AC012368.1
ENSG00000272068	AL365181.2
ENSG00000132970	WASF3
ENSG00000231940	RPS7P3
ENSG00000143365	RORC
ENSG00000101670	LIPG
ENSG00000275025	AC002401.3
ENSG00000259706	HSP90B2P
ENSG00000118526	TCF21
ENSG00000103811	CTSH
ENSG00000276418	AC036214.3
ENSG00000263155	MYZAP
ENSG00000114541	FRMD4B
ENSG00000105963	ADAP1
ENSG00000056736	IL17RB
ENSG00000126562	WNK4
ENSG00000183935	HTR7P1
ENSG00000169220	RGS14
ENSG00000197635	DPP4
ENSG00000214900	LINC01588
ENSG00000134343	ANO3
ENSG00000153086	ACMSD
ENSG00000128045	RASL11B
ENSG00000177133	LINC00982
ENSG00000070759	TESK2
ENSG00000136159	NUDT15
ENSG00000214290	COLCA2
ENSG00000197847	SLC22A20
ENSG00000124915	DKFZP434K028
ENSG00000141448	GATA6
ENSG00000069535	MAOB
ENSG00000276107	AC037198.2
ENSG00000104267	CA2
ENSG00000187676	B3GLCT
ENSG00000176124	DLEU1
ENSG00000019485	PRDM11
ENSG00000279968	GVQW2

ENSG00000139132	FGD4
ENSG00000168675	LDLRAD4
ENSG00000197837	HIST4H4
ENSG00000229097	CALM2P2
ENSG00000099958	DERL3
ENSG00000259426	AC027237.3
ENSG00000173947	PIFO
ENSG00000261270	AC012181.3
ENSG00000275410	HNF1B
ENSG00000248596	AC139491.2
ENSG00000130052	STARD8
ENSG00000165795	NDRG2
ENSG00000161921	CXCL16
ENSG00000102471	NDFIP2
ENSG00000137251	TINAG
ENSG00000261618	AC083837.1
ENSG00000152413	HOMER1
ENSG00000129654	FOXJ1
ENSG00000125845	BMP2
ENSG00000163141	BNIPL
ENSG00000160703	NLRX1
ENSG00000134574	DDB2
ENSG00000254165	AC090739.1
ENSG00000083535	PIBF1
ENSG00000121690	DEPDC7
ENSG00000136111	TBC1D4
ENSG00000215217	C5orf49
ENSG00000136040	PLXNC1
ENSG00000011105	TSPAN9
ENSG00000148655	LRMDA
ENSG00000233025	CRYZP1
ENSG00000260669	AL096870.2
ENSG00000119946	CNNM1
ENSG00000236883	AP001615.1
ENSG00000254911	SCARNA9
ENSG00000175832	ETV4
ENSG00000080293	SCTR
ENSG00000254101	LINC02055
ENSG00000165269	AQP7
ENSG00000168743	NPNT
ENSG00000133104	SPART
ENSG00000224417	AL606970.1
ENSG00000170545	SMAGP
ENSG00000256802	AC022613.1
ENSG00000167536	DHRS13

ENSG00000241404	EGFL8
ENSG00000101115	SALL4
ENSG00000241570	PAQR9-AS1
ENSG00000255750	AC022509.1
ENSG00000165923	AGBL2
ENSG00000100739	BDKRB1
ENSG00000088881	EBF4
ENSG00000186105	LRRC70
ENSG00000121671	CRY2
ENSG00000197748	CFAP43
ENSG00000123358	NR4A1
ENSG00000116396	KCNC4
ENSG00000178026	LRRC75B
ENSG00000153832	FBXO36
ENSG00000167771	RCOR2
ENSG00000173588	CEP83
ENSG00000091513	TF
ENSG00000100196	KDELR3
ENSG00000088926	F11
ENSG00000111199	TRPV4
ENSG00000138111	MFSD13A
ENSG00000162772	ATF3
ENSG00000116679	IVNS1ABP
ENSG00000118707	TGIF2
ENSG00000121039	RDH10
ENSG00000235162	C12orf75
ENSG00000117360	PRPF3
ENSG00000111249	CUX2
ENSG00000117595	IRF6
ENSG00000198168	SVIP
ENSG00000273523	AL139082.1
ENSG00000255970	LINC02421
ENSG00000274776	AC090241.3
ENSG00000279481	AC104791.2
ENSG00000237115	AL139805.2
ENSG00000198221	AFDN-AS1
ENSG00000279058	AGAP14
ENSG00000278967	AL138847.2
ENSG00000230328	AL359893.1
ENSG00000234232	AC243772.3
ENSG00000101342	TLDC2
ENSG00000213085	CFAP45
ENSG00000279148	AC126474.1
ENSG00000246095	LINC01096
ENSG00000280387	AL109806.1

ENSG00000235554	AC005822.1
ENSG00000198133	TMEM229B
ENSG00000248492	ZFAT-AS1
ENSG00000234750	AC012618.2
ENSG00000226318	RPS3AP38
ENSG00000232859	LYRM9
ENSG00000277589	AC244093.4
ENSG00000232926	AC000078.1
ENSG00000265907	AP000919.2
ENSG00000279949	AC022784.8
ENSG00000114993	RTKN
ENSG00000269068	AC009955.4
ENSG00000272102	AL133406.3
ENSG00000241158	ADAMTS9-AS1
ENSG00000253428	LINC01942
ENSG00000276434	AL136221.1
ENSG00000260808	AP003096.1
ENSG00000138606	SHF
ENSG00000268520	AC008750.5
ENSG00000254388	DUTP2
ENSG00000278022	AC118658.2
ENSG00000262172	AC116025.2
ENSG00000227279	AC110015.1
ENSG00000263466	AC006441.1
ENSG00000276603	AL109614.1
ENSG00000227008	AL009174.1
ENSG00000260279	AC137932.1
ENSG00000256664	AC025423.2
ENSG00000174032	SLC25A30
ENSG00000225951	ODF2-AS1
ENSG00000105672	ETV2
ENSG00000161647	MPP3
ENSG00000222898	RN7SKP97
ENSG00000149300	C11orf52
ENSG00000136122	BORA
ENSG00000166341	DCHS1
ENSG00000102048	ASB9
ENSG00000263745	AP005230.1
ENSG00000156711	MAPK13
ENSG00000100218	RSPH14
ENSG00000241388	HNF1A-AS1
ENSG00000264058	AC073508.2
ENSG00000135100	HNF1A
ENSG00000174804	FZD4
ENSG00000138587	MNS1

ENSG00000132464	ENAM
ENSG00000119125	GDA
ENSG00000112320	SOBP
ENSG00000176383	B3GNT4
ENSG00000108381	ASPA
ENSG00000259120	SMIM6
ENSG00000223572	CKMT1A
ENSG00000110344	UBE4A
ENSG00000139737	SLAIN1
ENSG00000093134	VNN3
ENSG00000182054	IDH2
ENSG00000174099	MSRB3
ENSG00000135218	CD36
ENSG00000235016	SEMA3F-AS1
ENSG00000204516	MICB
ENSG00000121057	AKAP1
ENSG00000259485	LINC02253
ENSG00000173198	CYSLTR1
ENSG00000120688	WBP4
ENSG00000165807	PPP1R36
ENSG00000137656	BUD13
ENSG00000141741	MIEN1
ENSG00000173653	RCE1
ENSG00000142798	HSPG2
ENSG00000111716	LDHB
ENSG00000227354	RBM26-AS1
ENSG00000188522	FAM83G
ENSG00000245385	AP003396.1
ENSG00000196411	EPHB4
ENSG00000236526	AL035448.1
ENSG00000120690	ELF1
ENSG00000237938	AL450998.3
ENSG00000177173	NAP1L4P1
ENSG00000164050	PLXNB1
ENSG00000274253	AC138649.1
ENSG00000203999	LINC01270
ENSG00000089505	CMTM1
ENSG00000070610	GBA2
ENSG00000159713	TPPP3
ENSG00000270149	AL591806.3
ENSG00000117122	MFAP2
ENSG00000251414	AC138956.2
ENSG00000170089	AC106795.1
ENSG00000149503	INCENP
ENSG00000172977	KAT5

ENSG00000136883	KIF12
ENSG00000136146	MED4
ENSG00000238197	PAXBP1-AS1
ENSG00000124782	RREB1
ENSG00000157557	ETS2
ENSG00000178445	GLDC
ENSG00000162105	SHANK2
ENSG00000162174	ASRGL1
ENSG00000134853	PDGFRA
ENSG00000175634	RPS6KB2
ENSG00000198824	CHAMP1
ENSG00000076826	CAMSAP3
ENSG00000107185	RGP1
ENSG00000111057	KRT18
ENSG00000205710	C17orf107
ENSG00000277639	AC007906.2
ENSG00000120697	ALG5
ENSG00000205436	EXOC3L4
ENSG00000139746	RBM26
ENSG00000271781	AC026740.1
ENSG00000184716	SERINC4
ENSG00000125246	CLYBL
ENSG00000183161	FANCF
ENSG00000100078	PLA2G3
ENSG00000102531	FNDC3A
ENSG00000260686	AC008669.1
ENSG00000187742	SECISBP2
ENSG00000107104	KANK1
ENSG00000183395	PMCH
ENSG00000147180	ZNF711
ENSG00000248124	RRN3P1
ENSG00000272604	AC073073.2
ENSG00000204428	LY6G5C
ENSG00000075856	SART3
ENSG00000087152	ATXN7L3
ENSG00000196656	AC004057.1
ENSG00000092850	TEKT2
ENSG00000168061	SAC3D1
ENSG00000070047	PHRF1
ENSG00000278977	AC003101.3
ENSG00000054690	PLEKHH1
ENSG00000116661	FBXO2
ENSG00000139354	GAS2L3
ENSG00000077514	POLD3
ENSG00000174243	DDX23

ENSG00000139117	CPNE8
ENSG00000276550	HERC2P2
ENSG00000108592	FTSJ3
ENSG00000163050	COQ8A
ENSG00000085999	RAD54L
ENSG00000198944	SOWAHA
ENSG00000204420	MPIG6B
ENSG00000187091	PLCD1
ENSG00000167543	TP53I13
ENSG00000239827	SUGT1P3
ENSG00000113194	FAF2
ENSG00000224078	SNHG14
ENSG00000228696	ARL17B
ENSG00000091073	DTX2
ENSG00000165458	INPPL1
ENSG00000186088	GSAP
ENSG00000085185	BCORL1
ENSG00000150401	DCUN1D2
ENSG00000140474	ULK3
ENSG00000100557	C14orf105
ENSG00000139835	GRTP1
ENSG00000163739	CXCL1
ENSG00000278616	BEND3P3
ENSG00000165195	PIGA
ENSG00000080618	CPB2
ENSG00000167861	HID1
ENSG00000121310	ECHDC2
ENSG00000182950	ODF3L1
ENSG00000096696	DSP
ENSG00000204610	TRIM15
ENSG00000129007	CALML4
ENSG00000066468	FGFR2
ENSG00000101160	CTSZ
ENSG00000166689	PLEKHA7
ENSG00000238083	LRRC37A2
ENSG00000198742	SMURF1
ENSG00000120885	CLU
ENSG00000174233	ADCY6
ENSG00000255164	AF235103.1
ENSG00000091262	ABCC6
ENSG00000170364	SETMAR
ENSG00000170927	PKHD1
ENSG00000257181	AC025423.4
ENSG00000169551	CT55
ENSG00000134146	DPH6

ENSG00000183421	RIPK4
ENSG00000030066	NUP160
ENSG00000140543	DET1
ENSG00000108830	RND2
ENSG00000121454	LHX4
ENSG00000138835	RGS3
ENSG00000117425	PTCH2
ENSG00000221829	FANCG
ENSG00000166435	XRRA1
ENSG00000165280	VCP
ENSG00000248643	RBM14-RBM4
ENSG00000148057	IDNK
ENSG00000110274	CEP164
ENSG00000261845	AC124283.1
ENSG00000189050	RNFT1
ENSG00000171617	ENC1
ENSG00000154080	CHST9
ENSG00000118058	KMT2A
ENSG00000180891	CUEDC1
ENSG00000104635	SLC39A14
ENSG00000126746	ZNF384
ENSG00000170421	KRT8
ENSG00000152193	RNF219
ENSG00000206149	HERC2P9
ENSG00000173930	SLCO4C1
ENSG00000053328	METTL24
ENSG00000102038	SMARCA1
ENSG00000226015	CCT8P1
ENSG00000166439	RNF169
ENSG00000262904	TMPOP2
ENSG00000213638	ADAT3
ENSG00000232729	AC211433.1
ENSG00000170113	NIPA1
ENSG00000143674	MAP3K21
ENSG00000168876	ANKRD49
ENSG00000140832	MARVELD3
ENSG00000104643	MTMR9
ENSG00000132740	IGHMBP2
ENSG00000240429	LRRFIP1P1
ENSG00000160584	SIK3
ENSG00000183020	AP2A2
ENSG00000142186	SCYL1
ENSG00000124602	UNC5CL
ENSG00000104998	IL27RA
ENSG00000166676	TVP23A

ENSG00000276101	AC027601.4
ENSG00000120738	EGR1
ENSG00000168938	PPIC
ENSG00000092445	TYRO3
ENSG00000174428	GTF2IRD2B
ENSG00000243696	AC006254.1
ENSG00000114315	HES1
ENSG00000175581	MRPL48
ENSG00000119630	PGF
ENSG00000139613	SMARCC2
ENSG00000136478	TEX2
ENSG00000237976	AL391069.3
ENSG00000141736	ERBB2
ENSG00000273274	ZBTB8B
ENSG00000185829	ARL17A
ENSG00000139323	POC1B
ENSG00000090382	LYZ
ENSG00000086848	ALG9
ENSG00000163399	ATP1A1
ENSG00000132640	BTBD3
ENSG00000248508	SRP14-AS1
ENSG00000072609	CHFR
ENSG00000109118	PHF12
ENSG00000174500	GCSAM
ENSG00000113249	HAVCR1
ENSG00000228434	AC004951.1
ENSG00000172830	SSH3
ENSG00000281571	AC241585.3
ENSG00000164344	KLKB1
ENSG00000204147	ASAH2B
ENSG00000112419	PHACTR2
ENSG00000136490	LIMD2
ENSG00000005156	LIG3
ENSG00000235522	AC010978.1
ENSG00000087448	KLHL42
ENSG00000250337	LINC01021
ENSG00000267041	ZNF850
ENSG00000130396	AFDN
ENSG00000250696	AC111000.4
ENSG00000104691	UBXN8
ENSG00000095539	SEMA4G
ENSG00000135679	MDM2
ENSG00000141338	ABCA8
ENSG00000267009	AC007780.1
ENSG00000183785	TUBA8

ENSG00000165916	PSMC3
ENSG00000254665	AC091053.1
ENSG00000253797	UTP14C
ENSG00000116741	RGS2
ENSG00000245848	CEBPA
ENSG00000157350	ST3GAL2
ENSG00000253719	ATXN7L3B
ENSG00000005486	RHBDD2
ENSG00000253305	PCDHGB6
ENSG00000142611	PRDM16
ENSG00000004864	SLC25A13
ENSG00000137700	SLC37A4
ENSG00000117707	PROX1
ENSG00000259417	CTXND1
ENSG00000276073	AL034549.2
ENSG00000214425	LRRC37A4P
ENSG00000165118	C9orf64
ENSG00000198814	GK
ENSG00000134780	DAGLA
ENSG00000237152	DLEU7-AS1
ENSG00000079950	STX7
ENSG00000196118	CCDC189
ENSG00000079739	PGM1
ENSG00000232906	AC092685.1
ENSG00000243775	OSTCP1
ENSG00000111859	NEDD9
ENSG00000149823	VPS51
ENSG00000131096	PYY
ENSG00000168781	PPIP5K1
ENSG00000103067	ESRP2
ENSG00000142789	CELA3A
ENSG00000102753	KPNA3
ENSG00000145198	VWA5B2
ENSG00000184992	BRI3BP
ENSG00000126749	EMG1
ENSG00000277459	AP001527.2
ENSG00000126759	CFP
ENSG00000173120	KDM2A
ENSG00000259539	AC051619.6
ENSG00000142530	FAM71E1
ENSG00000253875	AC013643.2
ENSG00000241886	AC112496.1
ENSG00000258815	AC131532.1
ENSG00000234518	PTGES3P1
ENSG00000224468	AL354953.1

ENSG00000183963	SMTN
ENSG00000135587	SMPD2
ENSG00000146083	RNF44
ENSG00000196123	KIAA0895L
ENSG00000141349	G6PC3
ENSG00000275576	AL049539.1
ENSG00000264608	AC005726.4
ENSG00000165113	GKAP1
ENSG00000257727	CNPY2
ENSG00000076604	TRAF4
ENSG00000247796	AC008966.1
ENSG00000225492	GBP1P1
ENSG00000102996	MMP15
ENSG00000167985	SDHAF2
ENSG00000166444	ST5
ENSG00000188092	GPR89B
ENSG00000111834	RSPH4A
ENSG00000186998	EMID1
ENSG00000076641	PAG1
ENSG00000176681	LRRC37A
ENSG00000257315	ZBED6
ENSG00000196458	ZNF605
ENSG00000279059	AC007485.2
ENSG00000159082	SYNJ1
ENSG00000182118	FAM89A
ENSG00000125968	ID1
ENSG00000121481	RNF2
ENSG00000107341	UBE2R2
ENSG00000213619	NDUFS3
ENSG00000205352	PRR13
ENSG00000257386	AC025257.1
ENSG00000077458	FAM76B
ENSG00000257550	AC023509.2
ENSG00000008323	PLEKHG6
ENSG00000177830	CHID1
ENSG00000181827	RFX7
ENSG00000110514	MADD
ENSG00000137713	PPP2R1B
ENSG00000140406	TLNRD1
ENSG00000094914	AAAS
ENSG00000138759	FRAS1
ENSG00000107338	SHB
ENSG00000146409	SLC18B1
ENSG00000149792	MRPL49
ENSG00000068097	HEATR6

ENSG00000135124	P2RX4
ENSG00000242028	HYPK
ENSG00000111602	TIMELESS
ENSG00000102978	POLR2C
ENSG00000102034	ELF4
ENSG00000101384	JAG1
ENSG00000226137	BAIAP2-AS1
ENSG00000249786	EAF1-AS1
ENSG00000149948	HMGA2
ENSG00000283154	IQCJ-SCHIP1
ENSG00000064787	BCAS1
ENSG00000269821	KCNQ1OT1
ENSG00000140400	MAN2C1
ENSG00000275835	TUBGCP5
ENSG00000149554	CHEK1
ENSG00000254873	AP001267.1
ENSG00000087074	PPP1R15A
ENSG00000227372	TP73-AS1
ENSG00000197044	ZNF441
ENSG00000234222	LIX1L-AS1
ENSG00000010539	ZNF200
ENSG00000230185	C9orf147
ENSG00000102003	SYP
ENSG00000136141	LRCH1
ENSG00000136108	CKAP2
ENSG00000111642	CHD4
ENSG00000260914	AC026464.4
ENSG00000159063	ALG8
ENSG00000184056	VPS33B
ENSG00000110048	OSBP
ENSG00000223773	CD99P1
ENSG00000272755	AC245297.2
ENSG00000197892	KIF13B
ENSG00000119888	EPCAM
ENSG00000137075	RNF38
ENSG00000108826	MRPL27
ENSG00000106541	AGR2
ENSG00000255874	LINC00346
ENSG00000079459	FDFT1
ENSG00000071243	ING3
ENSG00000144152	FBLN7
ENSG00000253535	AC120193.1
ENSG00000162086	ZNF75A
ENSG00000089902	RCOR1
ENSG00000170145	SIK2

ENSG00000165730	STOX1
ENSG00000198722	UNC13B
ENSG00000162073	PAQR4
ENSG00000198056	PRIM1
ENSG00000074266	EED
ENSG00000104129	DNAJC17
ENSG00000117791	MARC2
ENSG00000185324	CDK10
ENSG00000231185	SPRY4-AS1
ENSG00000173894	CBX2
ENSG00000140521	POLG
ENSG00000073584	SMARCE1
ENSG00000137073	UBAP2
ENSG00000085276	MECOM
ENSG00000166483	WEE1
ENSG00000139436	GIT2
ENSG00000170242	USP47
ENSG00000180525	PRR26
ENSG00000109685	NSD2
ENSG00000102554	KLF5
ENSG00000140525	FANCI
ENSG00000108187	PBLD
ENSG00000091583	APOH
ENSG00000168005	C11orf84
ENSG00000135048	TMEM2
ENSG00000109181	UGT2B10
ENSG00000256340	ABCC6P1
ENSG00000136156	ITM2B
ENSG00000121058	COIL
ENSG00000121073	SLC35B1
ENSG00000278615	C11orf98
ENSG00000279953	AC117503.4
ENSG00000151498	ACAD8
ENSG00000213462	ERV3-1
ENSG00000230928	AL139241.1
ENSG00000132286	TIMM10B
ENSG00000267040	AC027097.1
ENSG00000073605	GSDMB
ENSG00000173175	ADCY5
ENSG00000166831	RBPMS2
ENSG00000172932	ANKRD13D
ENSG00000185480	PARPBP
ENSG00000126602	TRAP1
ENSG00000065675	PRKCQ
ENSG00000229931	AL137003.1

ENSG00000109339	MAPK10
ENSG00000135392	DNAJC14
ENSG00000163879	DNALI1
ENSG00000173327	MAP3K11
ENSG00000149292	TTC12
ENSG00000182934	SRPRA
ENSG00000125447	GGA3
ENSG00000226232	NPIPB14P
ENSG00000111647	UHRF1BP1L
ENSG00000254417	ANO1-AS2
ENSG00000129173	E2F8
ENSG00000151366	NDUFC2
ENSG00000183354	KIAA2026
ENSG00000167553	TUBA1C
ENSG00000171234	UGT2B7
ENSG00000125319	C17orf53
ENSG00000177084	POLE
ENSG00000140279	DUOX2
ENSG00000167799	NUDT8
ENSG00000084453	SLCO1A2
ENSG00000198416	ZNF658B
ENSG00000185418	TARSL2
ENSG00000087095	NLK
ENSG00000244045	TMEM199
ENSG00000148400	NOTCH1
ENSG00000259781	HMGB1P6
ENSG00000204536	CCHCR1
ENSG00000158163	DZIP1L
ENSG00000274021	AC024909.3
ENSG00000257764	AC020656.1
ENSG00000136636	KCTD3
ENSG00000214087	ARL16
ENSG00000159217	IGF2BP1
ENSG00000119632	IFI27L2
ENSG00000139318	DUSP6
ENSG00000111667	USP5
ENSG00000142871	CYR61
ENSG00000165121	AL353743.1
ENSG00000160201	U2AF1
ENSG00000241889	AC079944.2
ENSG00000162520	SYNC
ENSG00000130770	ATPIF1
ENSG00000109705	NKX3-2
ENSG00000204444	APOM
ENSG00000108861	DUSP3

ENSG00000109046	WSB1
ENSG00000101856	PGRMC1
ENSG00000104147	OIP5
ENSG00000261408	TEN1-CDK3
ENSG00000263001	GTF2I
ENSG00000175220	ARHGAP1
ENSG00000125676	THOC2
ENSG00000160602	NEK8
ENSG00000187778	MCRS1
ENSG00000139428	MMAB
ENSG00000254469	AP002495.1
ENSG00000143869	GDF7
ENSG00000153714	LURAP1L
ENSG00000280010	AP001350.2
ENSG00000174231	PRPF8
ENSG00000248445	SEMA6A-AS1
ENSG00000164715	LMTK2
ENSG00000087087	SRRT
ENSG00000178301	AQP11
ENSG00000119720	NRDE2
ENSG00000233984	RPSAP14
ENSG00000121350	PYROXD1
ENSG00000131871	SELENOS
ENSG00000169242	EFNA1
ENSG00000135390	ATP5G2
ENSG00000123444	KBTBD4
ENSG00000103274	NUBP1
ENSG00000172409	CLP1
ENSG00000114841	DNAH1
ENSG00000185813	PCYT2
ENSG00000139579	NABP2
ENSG00000174871	CNIH2
ENSG00000167283	ATP5L
ENSG00000153404	PLEKHG4B
ENSG00000115935	WIPF1
ENSG00000116128	BCL9
ENSG00000223855	HRAT92
ENSG00000110075	PPP6R3
ENSG00000188338	SLC38A3
ENSG00000159259	CHAF1B
ENSG00000263096	AC007638.2
ENSG00000180884	ZNF792
ENSG00000272491	AL109659.2
ENSG00000157450	RNF111
ENSG00000260118	AL157700.1

ENSG00000250848	AC021087.2
ENSG00000102786	INTS6
ENSG00000236581	STARD13-AS
ENSG00000099869	IGF2-AS
ENSG00000250569	NTAN1P2
ENSG00000112305	SMAP1
ENSG00000173517	PEAK1
ENSG00000137802	MAPKBP1
ENSG00000035499	DEPDC1B
ENSG00000170468	RIOX1
ENSG00000073060	SCARB1
ENSG00000275854	AC084824.4
ENSG00000267303	AC011511.4
ENSG00000118523	CTGF
ENSG00000139546	TARBP2
ENSG00000113205	PCDHB3
ENSG00000178409	BEND3
ENSG00000175216	CKAP5
ENSG00000110911	SLC11A2
ENSG00000164045	CDC25A
ENSG00000271843	AC012557.1
ENSG00000248334	WHAMMP2
ENSG00000158156	XKR8
ENSG00000014138	POLA2
ENSG00000277147	LINC00869
ENSG00000119714	GPR68
ENSG00000137822	TUBGCP4
ENSG00000155090	KLF10
ENSG00000159086	PAXBP1
ENSG00000119139	TJP2
ENSG00000114019	AMOTL2
ENSG00000226644	AL121899.1
ENSG00000066084	DIP2B
ENSG00000106686	SPATA6L
ENSG00000173456	RNF26
ENSG00000113048	MRPS27
ENSG00000261098	AP000766.1
ENSG00000076053	RBM7
ENSG00000104218	CSPP1
ENSG00000070540	WIPI1
ENSG00000184445	KNTC1
ENSG00000253669	AP003356.1
ENSG00000087299	L2HGDH
ENSG00000197457	STMN3
ENSG00000069956	MAPK6

ENSG00000267648	AC060766.5
ENSG00000213514	AL731556.1
ENSG00000280038	DNM1P41
ENSG00000129317	PUS7L
ENSG00000131475	VPS25
ENSG00000275512	AC007998.4
ENSG00000175376	EIF1AD
ENSG00000184925	LCN12
ENSG00000165338	HECTD2
ENSG00000150712	MTMR12
ENSG00000159423	ALDH4A1
ENSG00000259884	AC025259.3
ENSG00000110958	PTGES3
ENSG00000175414	ARL10
ENSG00000135387	CAPRIN1
ENSG00000094916	CBX5
ENSG00000165475	CRYL1
ENSG00000214826	DDX12P
ENSG00000166881	NEMP1
ENSG00000139344	AMDHD1
ENSG00000253250	C8orf88
ENSG00000113648	H2AFY
ENSG00000179388	EGR3
ENSG00000112039	FANCE
ENSG00000144589	STK11IP
ENSG00000240694	PNMA2
ENSG00000169230	PRELID1
ENSG00000100031	GGT1
ENSG00000123908	AGO2
ENSG00000114383	TUSC2
ENSG00000110321	EIF4G2
ENSG00000105219	CNTD2
ENSG00000065029	ZNF76
ENSG00000189079	ARID2
ENSG00000149357	LAMTOR1
ENSG00000196233	LCOR
ENSG00000253200	AC037459.3
ENSG00000177106	EPS8L2
ENSG00000171316	CHD7
ENSG00000103932	RPAP1
ENSG00000196793	ZNF239
ENSG00000161914	ZNF653
ENSG00000081791	KIAA0141
ENSG00000095464	PDE6C
ENSG00000090621	PABPC4

ENSG00000105135	ILVBL
ENSG00000136158	SPRY2
ENSG00000134986	NREP
ENSG00000259577	CERNA1
ENSG00000145087	STXBP5L
ENSG00000131781	FMO5
ENSG00000140465	CYP1A1
ENSG00000167216	KATNAL2
ENSG00000278867	AC090616.7
ENSG00000183617	MRPL54
ENSG00000118363	SPCS2
ENSG00000279522	AC008536.3
ENSG00000136014	USP44
ENSG00000197647	ZNF433
ENSG00000143375	CGN
ENSG00000277972	CISD3
ENSG00000113658	SMAD5
ENSG00000157999	ANKRD61
ENSG00000106603	COA1
ENSG00000160447	PKN3
ENSG00000280649	AC245100.8
ENSG00000204282	TNRC6C-AS1
ENSG00000102796	DHRS12
ENSG00000095066	HOOK2
ENSG00000166337	TAF10
ENSG00000166004	CEP295
ENSG00000158042	MRPL17
ENSG00000152270	PDE3B
ENSG00000106261	ZKSCAN1
ENSG00000100982	PCIF1
ENSG00000165948	IFI27L1
ENSG00000110871	COQ5
ENSG00000130584	ZBTB46
ENSG00000135486	HNRNPA1
ENSG00000273749	CYFIP1
ENSG00000275601	AC011330.2
ENSG00000186185	KIF18B
ENSG00000274403	AC090510.2
ENSG00000133703	KRAS
ENSG00000250508	AP000808.1
ENSG00000106258	CYP3A5
ENSG00000087842	PIR
ENSG00000279594	AL049780.3
ENSG00000242779	ZNF702P
ENSG00000179950	PUF60

ENSG00000053702	NRIP2
ENSG00000158555	GDPD5
ENSG00000111731	C2CD5
ENSG00000130675	MNX1
ENSG00000181690	PLAG1
ENSG00000259494	MRPL46
ENSG00000274281	AC022929.2
ENSG00000147526	TACC1
ENSG00000167325	RRM1
ENSG00000215788	TNFRSF25
ENSG00000196696	PDXDC2P-NPIPB14P
ENSG00000143845	ETNK2
ENSG00000167525	PROCA1
ENSG00000170085	SIMC1
ENSG00000099814	CEP170B
ENSG00000152382	TADA1
ENSG00000110429	FBXO3
ENSG00000086827	ZW10
ENSG00000176907	C8orf4
ENSG00000135404	CD63
ENSG00000174903	RAB1B
ENSG00000080493	SLC4A4
ENSG00000123119	NECAB1
ENSG00000243566	UPK3B
ENSG00000179604	CDC42EP4
ENSG00000134825	TMEM258
ENSG00000140259	MFAP1
ENSG00000147854	UHRF2
ENSG00000013573	DDX11
ENSG00000197256	KANK2
ENSG00000162227	TAF6L
ENSG00000104064	GABPB1
ENSG00000178927	C17orf62
ENSG00000198774	RASSF9
ENSG00000157240	FZD1
ENSG00000044459	CNTLN
ENSG00000204172	AGAP9
ENSG00000055163	CYFIP2
ENSG00000140548	ZNF710
ENSG00000140553	UNC45A
ENSG00000188070	C11orf95
ENSG00000117480	FAAH
ENSG00000167965	MLST8
ENSG00000137880	GCHFR
ENSG00000166938	DIS3L

ENSG00000110492	MDK
ENSG00000171365	CLCN5
ENSG00000279019	AC009090.4
ENSG00000140367	UBE2Q2
ENSG00000275481	AC025031.4
ENSG00000206129	AC006305.1
ENSG00000160172	FAM86C2P
ENSG00000198826	ARHGAP11A
ENSG00000137216	TMEM63B
ENSG00000269743	SLC25A53
ENSG00000226416	MRPL23-AS1
ENSG00000016402	IL20RA
ENSG00000105699	LSR
ENSG00000114302	PRKAR2A
ENSG00000239306	RBM14
ENSG00000160293	VAV2
ENSG00000005075	POLR2J
ENSG00000109919	MTCH2
ENSG00000007866	TEAD3
ENSG00000073350	LLGL2
ENSG00000071539	TRIP13
ENSG00000159110	IFNAR2
ENSG00000133135	RNF128
ENSG00000145780	FEM1C
ENSG00000131979	GCH1
ENSG00000139719	VPS33A
ENSG00000155542	SETD9
ENSG00000112118	MCM3
ENSG00000254876	AL590705.5
ENSG00000081026	MAGI3
ENSG00000127586	CHTF18
ENSG00000271133	AC004130.1
ENSG00000182872	RBM10
ENSG00000265683	SYPL1P2
ENSG00000262576	PCDHGA4
ENSG00000132694	ARHGEF11
ENSG00000166562	SEC11C
ENSG00000147140	NONO
ENSG00000146707	POMZP3
ENSG00000170653	ATF7
ENSG00000277806	AC006213.4
ENSG00000127337	YEATS4
ENSG00000246451	AL049840.1
ENSG00000284128	AP000356.3
ENSG00000204356	NELFE

ENSG00000136811	ODF2
ENSG00000092931	MFSD11
ENSG00000157625	TAB3
ENSG00000267904	AC024075.1
ENSG00000141522	ARHGDIA
ENSG00000260804	LINC01963
ENSG00000110074	FOXRED1
ENSG00000235512	TAB3-AS2
ENSG00000100321	SYNGR1
ENSG00000116704	SLC35D1
ENSG00000197557	TTC30A
ENSG00000077684	JADE1
ENSG00000127666	TICAM1
ENSG00000269940	AL049840.3
ENSG00000165434	PGM2L1
ENSG00000156304	SCAF4
ENSG00000152404	CWF19L2
ENSG00000166333	ILK
ENSG00000165238	WNK2
ENSG00000236438	FAM157A
ENSG00000213930	GALT
ENSG00000018189	RUFY3
ENSG00000148943	LIN7C
ENSG00000122729	ACO1
ENSG00000085063	CD59
ENSG00000143442	POGZ
ENSG00000171792	RHNO1
ENSG00000284707	AC079781.5
ENSG00000108825	PTGES3L-AARSD1
ENSG00000168795	ZBTB5
ENSG00000054965	FAM168A
ENSG00000139531	SUOX
ENSG00000011114	BTBD7
ENSG00000108946	PRKAR1A
ENSG00000141232	TOB1
ENSG00000196091	MYBPC1
ENSG00000162009	SSTR5
ENSG00000176890	TYMS
ENSG00000137720	C11orf1
ENSG00000083642	PDS5B
ENSG00000126653	NSRP1
ENSG00000108854	SMURF2
ENSG00000262621	AC025283.2
ENSG00000143199	ADCY10
ENSG00000131462	TUBG1

ENSG00000197054	ZNF763
ENSG00000100479	POLE2
ENSG00000104957	CCDC130
ENSG00000277998	AC107075.1
ENSG00000198055	GRK6
ENSG00000068305	MEF2A
ENSG00000161642	ZNF385A
ENSG00000140326	CDAN1
ENSG00000105854	PON2
ENSG00000171236	LRG1
ENSG00000123353	ORMDL2
ENSG00000064607	SUGP2
ENSG00000280623	PCAT14
ENSG00000274349	ZNF658
ENSG00000275888	AC132872.3
ENSG00000111727	HCFC2
ENSG00000072786	STK10
ENSG00000204590	GNL1
ENSG00000109079	TNFAIP1
ENSG00000176732	PFN4
ENSG00000165804	ZNF219
ENSG00000182685	BRICD5
ENSG00000172058	SERF1A
ENSG00000064545	TMEM161A
ENSG00000051180	RAD51
ENSG00000136485	DCAF7
ENSG00000205517	RGL3
ENSG00000173486	FKBP2
ENSG00000136492	BRIP1
ENSG00000184863	RBM33
ENSG00000135452	TSPAN31
ENSG00000137871	ZNF280D
ENSG00000011258	MBTD1
ENSG00000128944	KNSTRN
ENSG00000183060	LYSMD4
ENSG00000042781	USH2A
ENSG00000163939	PBRM1
ENSG00000137055	PLAA
ENSG00000172354	GNB2
ENSG00000149499	EML3
ENSG00000138166	DUSP5
ENSG00000131242	RAB11FIP4
ENSG00000232748	AC135050.1
ENSG00000110090	CPT1A
ENSG00000101868	POLA1

ENSG00000163874	ZC3H12A
ENSG00000143815	LBR
ENSG00000137776	SLTM
ENSG00000165511	C10orf25
ENSG00000229980	TOB1-AS1
ENSG00000183684	ALYREF
ENSG00000213516	RBMXL1
ENSG00000110025	SNX15
ENSG00000147439	BIN3
ENSG00000137135	ARHGEF39
ENSG00000163719	MTMR14
ENSG00000135451	TROAP
ENSG00000146197	SCUBE3
ENSG00000015153	YAF2
ENSG00000143379	SETDB1
ENSG00000196814	MVB12B
ENSG00000228069	MTCO3P29
ENSG00000109072	VTN
ENSG00000141499	WRAP53
ENSG00000159388	BTG2
ENSG00000198830	HMGN2
ENSG00000062725	APPBP2
ENSG00000099849	RASSF7
ENSG00000025434	NR1H3
ENSG00000254996	ANKHD1-EIF4EBP3
ENSG00000196391	ZNF774
ENSG00000214717	ZBED1
ENSG00000152672	CLEC4F
ENSG00000263327	TAPT1-AS1
ENSG00000185347	C14orf80
ENSG00000154920	EME1
ENSG00000170537	TMC7
ENSG00000256667	KLRA1P
ENSG00000148985	PGAP2
ENSG00000125898	FAM110A
ENSG00000241468	ATP5J2
ENSG00000283375	AC087521.4
ENSG00000231752	EMBP1
ENSG00000129158	SERGEF
ENSG00000145781	COMMD10
ENSG00000163913	IFT122
ENSG00000140307	GTF2A2
ENSG00000175221	MED16
ENSG00000177685	CRACR2B
ENSG00000102103	PQBP1

ENSG00000095794	CREM
ENSG00000112309	B3GAT2
ENSG00000139679	LPAR6
ENSG00000011021	CLCN6
ENSG00000111788	AC009533.1
ENSG00000242299	AC073861.1
ENSG00000157110	RBPMS
ENSG00000188163	FAM166A
ENSG00000175265	GOLGA8A
ENSG00000186654	PRR5
ENSG00000141562	NARF
ENSG00000133731	IMPA1
ENSG00000151503	NCAPD3
ENSG00000112977	DAP
ENSG00000128739	SNRPN
ENSG00000267102	AC060766.1
ENSG00000151790	TDO2
ENSG00000145703	IQGAP2
ENSG00000204304	PBX2
ENSG00000172893	DHCR7
ENSG00000011422	PLAUR
ENSG00000167797	CDK2AP2
ENSG00000196418	ZNF124
ENSG00000267519	AC020916.1
ENSG00000169710	FASN
ENSG00000176986	SEC24C
ENSG00000176340	COX8A
ENSG00000233806	LINC01237
ENSG00000203804	ADAMTSL4-AS1
ENSG00000116580	GON4L
ENSG00000146828	SLC12A9
ENSG00000164466	SFXN1
ENSG00000014216	CAPN1
ENSG00000205531	NAP1L4
ENSG00000130699	TAF4
ENSG00000272888	LINC01578
ENSG00000124251	TP53TG5
ENSG00000196510	ANAPC7
ENSG00000137478	FCHSD2
ENSG00000102125	TAZ
ENSG00000197976	AKAP17A
ENSG00000236432	AC097662.1
ENSG00000168137	SETD5
ENSG00000136715	SAP130
ENSG00000186329	TMEM212

ENSG00000168806	LCMT2
ENSG00000139624	CERS5
ENSG00000189180	ZNF33A
ENSG00000162769	FLVCR1
ENSG00000124181	PLCG1
ENSG00000188785	ZNF548
ENSG00000186205	MARC1
ENSG00000137807	KIF23
ENSG00000148942	SLC5A12
ENSG00000161533	ACOX1
ENSG00000113318	MSH3
ENSG00000179889	PDXDC1
ENSG00000174827	PDZK1
ENSG00000266918	AC091132.4
ENSG00000166908	PIP4K2C
ENSG00000074603	DPP8
ENSG00000166387	PPFIBP2
ENSG00000198040	ZNF84
ENSG00000234338	AC073349.2
ENSG00000182473	EXOC7
ENSG00000224093	AL109613.1
ENSG00000246250	AC087521.2
ENSG00000188994	ZNF292
ENSG00000278540	ACACA
ENSG00000110218	PANX1
ENSG00000213079	SCAF8
ENSG00000133636	NTS
ENSG00000130005	GAMT
ENSG00000132773	TOE1
ENSG00000271793	AL589666.1
ENSG00000089248	ERP29
ENSG00000044446	PHKA2
ENSG00000172955	ADH6
ENSG00000108389	MTMR4
ENSG00000101444	AHCY
ENSG00000076382	SPAG5
ENSG00000281392	LINC00506
ENSG00000168517	HEXIM2
ENSG00000235236	AC137630.2
ENSG00000149089	APIP
ENSG00000258092	AC005841.1
ENSG00000168944	CEP120
ENSG00000099326	MZF1
ENSG00000214253	FIS1
ENSG00000004700	RECQL

ENSG00000169045	HNRNPH1
ENSG00000280798	LINC00294
ENSG00000056998	GYG2
ENSG00000269646	AC010487.2
ENSG00000266283	AC091588.3
ENSG00000127720	METTL25
ENSG00000163638	ADAMTS9
ENSG00000013523	ANGEL1
ENSG00000134070	IRAK2
ENSG00000181404	WASHC1
ENSG00000166228	PCBD1
ENSG00000100105	PATZ1
ENSG00000113212	PCDHB7
ENSG00000275066	SYNRG
ENSG00000144445	KANSL1L
ENSG00000029363	BCLAF1
ENSG00000143157	POGK
ENSG00000178950	GAK
ENSG00000168175	MAPK1IP1L
ENSG00000148200	NR6A1
ENSG00000183978	COA3
ENSG00000187699	C2orf88
ENSG00000187790	FANCM
ENSG00000065882	TBC1D1
ENSG00000238018	AC093110.1
ENSG00000179978	AC140134.1
ENSG00000259974	LINC00261
ENSG00000160888	IER2
ENSG00000164405	UQCRQ
ENSG00000213465	ARL2
ENSG00000183814	LIN9
ENSG00000279744	AC132938.6
ENSG00000166598	HSP90B1
ENSG00000253320	AZIN1-AS1
ENSG00000174574	AKIRIN1
ENSG00000278922	AC002310.6
ENSG00000138336	TET1
ENSG00000047634	SCML1
ENSG00000088451	TGDS
ENSG00000278311	GGNBP2
ENSG00000132478	UNK
ENSG00000249348	UGDH-AS1
ENSG00000183765	CHEK2
ENSG00000072422	RHOBTB1
ENSG00000131153	GINS2

ENSG00000076242	MLH1
ENSG00000177728	TMEM94
ENSG00000149923	PPP4C
ENSG00000178401	DNAJC22
ENSG00000196466	ZNF799
ENSG00000136770	DNAJC1
ENSG00000171634	BPTF
ENSG00000138867	GUCD1
ENSG00000077454	LRCH4
ENSG00000186723	OR10H1
ENSG00000103335	PIEZO1
ENSG00000278384	AL354822.1
ENSG00000089818	NECAP1
ENSG00000137513	NARS2
ENSG00000152795	HNRNPDL
ENSG00000168411	RFWD3
ENSG00000164211	STARD4
ENSG00000121621	KIF18A
ENSG00000092140	G2E3
ENSG00000132688	NES
ENSG00000128829	EIF2AK4
ENSG00000141505	ASGR1
ENSG00000256804	AC138466.2
ENSG00000120992	LYPLA1
ENSG00000164548	TRA2A
ENSG00000183458	AC138932.1
ENSG00000152518	ZFP36L2
ENSG00000140320	BAHD1
ENSG00000147535	PLPP5
ENSG00000276234	TADA2A
ENSG00000116138	DNAJC16
ENSG00000198324	FAM109A
ENSG00000140398	NEIL1
ENSG00000260941	LINC00622
ENSG00000176463	SLCO3A1
ENSG00000249437	NAIP
ENSG00000166582	CENPV
ENSG00000161955	TNFSF13
ENSG00000272269	AL138724.1
ENSG00000110711	AIP
ENSG00000111412	C12orf49
ENSG00000178623	GPR35
ENSG00000180210	F2
ENSG00000198858	R3HDM4
ENSG00000258017	AC011603.2

ENSG00000111605	CPSF6
ENSG00000166263	STXBP4
ENSG00000149308	NPAT
ENSG00000141582	CBX4
ENSG00000123191	ATP7B
ENSG00000143033	MTF2
ENSG00000105127	AKAP8
ENSG00000100906	NFKBIA
ENSG00000128191	DGCR8
ENSG00000133740	E2F5
ENSG00000238035	AC138035.1
ENSG00000088038	CNOT3
ENSG00000244480	AC005154.3
ENSG00000277837	AC090340.1
ENSG00000121741	ZMYM2
ENSG00000103199	ZNF500
ENSG00000023697	DERA
ENSG00000185262	UBALD2
ENSG00000257446	ZNF878
ENSG00000164011	ZNF691
ENSG00000125740	FOSB
ENSG00000153922	CHD1
ENSG00000114904	NEK4
ENSG00000257594	GALNT4
ENSG00000198157	HMGN5
ENSG00000242798	AC073842.2
ENSG00000090581	GNPTG
ENSG00000188559	RALGAPA2
ENSG00000197299	BLM
ENSG00000205423	CNEP1R1
ENSG00000140374	ETFA
ENSG00000215190	LINC00680
ENSG00000198677	TTC37
ENSG00000146433	TMEM181
ENSG00000197579	TOPORS
ENSG00000169750	RAC3
ENSG00000006282	SPATA20
ENSG00000178449	COX14
ENSG00000140319	SRP14
ENSG00000163378	EOGT
ENSG00000107758	PPP3CB
ENSG00000127663	KDM4B
ENSG00000120802	TMPO
ENSG00000109971	HSPA8
ENSG00000203865	ATP1A1-AS1

ENSG00000133704	IPO8
ENSG00000254422	AP000942.2
ENSG00000004534	RBM6
ENSG00000152939	MARVELD2
ENSG00000262160	AC020978.6
ENSG00000105855	ITGB8
ENSG00000232160	RAP2C-AS1
ENSG00000143367	TUFT1
ENSG00000145861	C1QTNF2
ENSG00000213585	VDAC1
ENSG00000231154	MORF4L2-AS1
ENSG00000123473	STIL
ENSG00000140262	TCF12
ENSG00000081041	CXCL2
ENSG00000187994	RINL
ENSG00000058804	NDC1
ENSG00000122882	ECD
ENSG00000255949	AP003419.1
ENSG00000184304	PRKD1
ENSG00000184545	DUSP8
ENSG00000130203	APOE
ENSG00000185049	NELFA
ENSG00000239883	PARGP1
ENSG00000259071	AL359397.2
ENSG00000272140	AC022400.5
ENSG00000134283	PPHLN1
ENSG00000166685	COG1
ENSG00000213753	CENPBD1P1
ENSG00000067369	TP53BP1
ENSG00000170325	PRDM10
ENSG00000241549	GUSBP2
ENSG00000010292	NCAPD2
ENSG00000036828	CASR
ENSG00000130940	CASZ1
ENSG00000196586	MYO6
ENSG00000130208	APOC1
ENSG00000132589	FLOT2
ENSG00000181991	MRPS11
ENSG00000165801	ARHGEF40
ENSG00000215193	PEX26
ENSG00000049656	CLPTM1L
ENSG00000011347	SYT7
ENSG00000138686	BBS7
ENSG00000010244	ZNF207
ENSG00000267598	AC011446.2

ENSG00000137100	DCTN3
ENSG00000110013	SIAE
ENSG00000033800	PIAS1
ENSG00000258366	RTEL1
ENSG00000168916	ZNF608
ENSG00000073792	IGF2BP2
ENSG00000169635	HIC2
ENSG00000173442	EHBP1L1
ENSG00000188107	EYS
ENSG00000099256	PRTFDC1
ENSG00000137497	NUMA1
ENSG00000144677	CTDSPL
ENSG00000157837	SPPL3
ENSG00000178425	NT5DC1
ENSG00000215301	DDX3X
ENSG00000139116	KIF21A
ENSG00000068796	KIF2A
ENSG00000214029	ZNF891
ENSG00000198938	MT-CO3
ENSG00000110171	TRIM3
ENSG00000011304	PTBP1
ENSG00000185624	P4HB
ENSG00000173218	VANGL1
ENSG00000170412	GPRC5C
ENSG00000183474	GTF2H2C
ENSG00000136381	IREB2
ENSG00000160191	PDE9A
ENSG00000134982	APC
ENSG00000182180	MRPS16
ENSG00000090686	USP48
ENSG00000090615	GOLGA3
ENSG00000204138	PHACTR4
ENSG00000249476	AC008467.1
ENSG00000263089	AC007114.2
ENSG00000132781	MUTYH
ENSG00000103876	FAH
ENSG00000122952	ZWINT
ENSG00000138614	INTS14
ENSG00000205758	CRYZL1
ENSG00000139218	SCAF11
ENSG00000103111	MON1B
ENSG00000188649	CC2D2B
ENSG00000144028	SNRNP200
ENSG00000101096	NFATC2
ENSG00000154240	CEP112

ENSG00000112242	E2F3
ENSG00000177932	ZNF354C
ENSG00000253731	PCDHGA6
ENSG00000137486	ARRB1
ENSG00000232300	FAM215B
ENSG00000156172	C8orf37
ENSG00000196204	RNF216P1
ENSG00000253485	PCDHGA5
ENSG00000077312	SNRPA
ENSG00000118507	AKAP7
ENSG00000177191	B3GNT8
ENSG00000111271	ACAD10
ENSG00000261063	AC009139.2
ENSG00000139990	DCAF5
ENSG00000117713	ARID1A
ENSG00000124171	PARD6B
ENSG00000270100	AC012065.4
ENSG00000105486	LIG1
ENSG00000196683	TOMM7
ENSG00000103978	TMEM87A
ENSG00000269556	TMEM185A
ENSG00000146826	C7orf43
ENSG00000241553	ARPC4
ENSG00000140043	PTGR2
ENSG00000180815	MAP3K15
ENSG00000145241	CENPC
ENSG00000279726	AC005609.5
ENSG00000173156	RHOD
ENSG00000205189	ZBTB10
ENSG00000255277	ABCC6P2
ENSG00000267281	AC023509.3
ENSG00000204604	ZNF468
ENSG00000225973	PIGBOS1
ENSG00000160783	PMF1
ENSG00000065328	MCM10
ENSG00000122971	ACADS
ENSG00000066583	ISOC1
ENSG00000198932	GPRASP1
ENSG00000166507	NDST2
ENSG00000140612	SEC11A
ENSG00000260853	AC109460.2
ENSG00000157600	TMEM164
ENSG00000168350	DEGS2
ENSG00000117228	GBP1
ENSG00000227671	AL390728.4

ENSG00000130695	CEP85
ENSG00000123124	WWP1
ENSG00000185219	ZNF445
ENSG00000147853	AK3
ENSG00000112293	GPLD1
ENSG00000090097	PCBP4
ENSG00000239945	AL627309.3
ENSG00000188229	TUBB4B
ENSG00000164776	PHKG1
ENSG00000105397	TYK2
ENSG00000122359	ANXA11
ENSG00000186448	ZNF197
ENSG00000241769	LINC00893
ENSG00000151748	SAV1
ENSG00000174021	GNG5
ENSG00000224578	HNRNPA1P48
ENSG00000197021	CXorf40B
ENSG00000280399	AC022497.1
ENSG00000233369	GTF2IP4
ENSG00000122986	HVCN1
ENSG00000111684	LPCAT3
ENSG00000259345	AC013652.1
ENSG00000141002	TCF25
ENSG00000230847	AC044797.1
ENSG00000260400	AL513534.1
ENSG00000253203	GUSBP3
ENSG00000132017	DCAF15
ENSG00000103671	TRIP4
ENSG00000133739	LRRCC1
ENSG00000128833	MYO5C
ENSG00000123219	CENPK
ENSG00000172340	SUCLG2
ENSG00000111652	COPS7A
ENSG00000261572	AC097639.1
ENSG00000265618	AC002094.2
ENSG00000157212	PAXIP1
ENSG00000234545	FAM133B
ENSG00000120129	DUSP1
ENSG00000189227	C15orf61
ENSG00000101745	ANKRD12
ENSG00000187678	SPRY4
ENSG00000111331	OAS3
ENSG00000213918	DNASE1
ENSG00000128989	ARPP19
ENSG00000235109	ZSCAN31

ENSG00000218891	ZNF579
ENSG00000281344	HELLPAR
ENSG00000198039	ZNF273
ENSG00000258738	AL121603.2
ENSG00000149922	TBX6
ENSG00000099783	HNRNPM
ENSG00000125863	MKKS
ENSG00000144741	SLC25A26
ENSG00000125834	STK35
ENSG00000169612	FAM103A1
ENSG00000185787	MORF4L1
ENSG00000172062	SMN1
ENSG00000249614	LINC02503
ENSG00000166224	SGPL1
ENSG00000129103	SUMF2
ENSG00000180773	SLC36A4
ENSG00000179922	ZNF784
ENSG00000198026	ZNF335
ENSG00000142856	ITGB3BP
ENSG00000161920	MED11
ENSG00000113141	IK
ENSG00000134575	ACP2
ENSG00000122877	EGR2
ENSG00000156970	BUB1B
ENSG00000188295	ZNF669
ENSG00000070010	UFD1
ENSG00000173905	GOLIM4
ENSG00000205269	TMEM170B
ENSG00000101193	GID8
ENSG00000012174	MBTPS2
ENSG00000079313	REXO1
ENSG00000101191	DIDO1
ENSG00000122678	POLM
ENSG00000132256	TRIM5
ENSG00000070081	NUCB2
ENSG00000237836	PHKA2-AS1
ENSG00000225663	MCRIP1
ENSG00000054148	PHPT1
ENSG00000101945	SUV39H1
ENSG00000163788	SNRK
ENSG00000259956	RBM15B
ENSG00000133997	MED6
ENSG00000132386	SERPINF1
ENSG00000160305	DIP2A
ENSG00000149609	C20orf144

ENSG00000198218	QRICH1
ENSG00000117569	PTBP2
ENSG00000188610	FAM72B
ENSG00000176868	AL358781.1
ENSG00000162825	NBPF20
ENSG00000139438	FAM222A
ENSG00000283235	AC139493.2
ENSG00000269226	TMSB15B
ENSG00000205581	HMGN1
ENSG00000122126	OCRL
ENSG00000160051	IQCC
ENSG00000158987	RAPGEF6
ENSG00000123416	TUBA1B
ENSG00000157181	C1orf27
ENSG00000179750	APOBEC3B
ENSG00000120805	ARL1
ENSG00000158470	B4GALT5
ENSG00000007520	TSR3
ENSG00000162063	CCNF
ENSG00000168152	THAP9
ENSG00000131351	HAUS8
ENSG00000173540	GMPPB
ENSG00000104375	STK3
ENSG00000101407	TTI1
ENSG00000123836	PFKFB2
ENSG00000188878	FBF1
ENSG00000152348	ATG10
ENSG00000113387	SUB1
ENSG00000143882	ATP6V1C2
ENSG00000259380	AC087473.1
ENSG00000166851	PLK1
ENSG00000169410	PTPN9
ENSG00000083828	ZNF586
ENSG00000233251	AC007743.1
ENSG00000182944	EWSR1
ENSG00000225484	NUTM2B-AS1
ENSG00000155008	APOOL
ENSG00000182095	TNRC18
ENSG00000180855	ZNF443
ENSG00000230724	LINC01001
ENSG00000169683	LRRC45
ENSG00000012232	EXTL3
ENSG00000046653	GPM6B
ENSG00000143315	PIGM
ENSG00000130227	XPO7

ENSG00000087586	AURKA
ENSG00000255561	FDXACB1
ENSG00000157881	PANK4
ENSG00000135930	EIF4E2
ENSG00000166415	WDR72
ENSG00000052126	PLEKHA5
ENSG00000257365	FNTB
ENSG00000164151	ICE1
ENSG00000151353	TMEM18
ENSG00000179943	FIZ1
ENSG00000143630	HCN3
ENSG00000173281	PPP1R3B
ENSG00000105289	TJP3
ENSG00000204209	DAXX
ENSG00000241685	ARPC1A
ENSG00000000457	SCYL3
ENSG00000116560	SFPQ
ENSG00000127483	HP1BP3
ENSG00000100380	ST13
ENSG00000168566	SNRNP48
ENSG00000096070	BRPF3
ENSG00000112759	SLC29A1
ENSG00000129003	VPS13C
ENSG00000214013	GANC
ENSG00000237773	AC003075.1
ENSG00000138778	CENPE
ENSG00000162645	GBP2
ENSG00000272918	AC005070.3
ENSG00000168246	UBTD2
ENSG00000181038	METTL23
ENSG00000140395	WDR61
ENSG00000136305	CIDEB
ENSG00000196911	KPNA5
ENSG00000103091	WDR59
ENSG00000167523	SPATA33
ENSG00000184675	AMER1
ENSG00000062194	GPBP1
ENSG00000197020	ZNF100
ENSG00000240731	AL139287.1
ENSG00000139641	ESYT1
ENSG00000122912	SLC25A16
ENSG00000132780	NASP
ENSG00000117625	RCOR3
ENSG00000159069	FBXW5
ENSG00000137288	UQCC2

ENSG00000151689	INPP1
ENSG00000145833	DDX46
ENSG00000169371	SNUPN
ENSG00000103495	MAZ
ENSG00000124160	NCOA5
ENSG00000268565	AC005339.1
ENSG00000114739	ACVR2B
ENSG00000111196	MAGOHB
ENSG00000015171	ZMYND11
ENSG00000143158	MPC2
ENSG00000224975	INE1
ENSG00000001631	KRIT1
ENSG00000158079	PTPDC1
ENSG00000184990	SIVA1
ENSG00000070761	CFAP20
ENSG00000069248	NUP133
ENSG00000161800	RACGAP1
ENSG00000167196	FBXO22
ENSG00000267632	AC067852.6
ENSG00000165525	NEMF
ENSG00000129128	SPCS3
ENSG00000188486	H2AFX
ENSG00000215105	TTC3P1
ENSG00000025156	HSF2
ENSG00000257949	TEN1
ENSG00000182749	PAQR7
ENSG00000197965	MPZL1
ENSG00000168118	RAB4A
ENSG00000214046	SMIM7
ENSG00000149269	PAK1
ENSG00000096063	SRPK1
ENSG00000181826	RELL1
ENSG00000159459	UBR1
ENSG00000137331	IER3
ENSG00000278769	AC090510.3
ENSG00000046651	OFD1
ENSG00000151657	KIN
ENSG00000076924	XAB2
ENSG00000160404	TOR2A
ENSG00000119682	AREL1
ENSG00000130023	ERMARD
ENSG00000204152	TIMM23B
ENSG00000152818	UTRN
ENSG00000279069	AC015813.5
ENSG00000152457	DCLRE1C

ENSG00000156876	SASS6
ENSG00000197472	ZNF695
ENSG00000135775	COG2
ENSG00000083896	YTHDC1
ENSG00000178966	RMI1
ENSG00000116273	PHF13
ENSG00000104889	RNASEH2A
ENSG00000166510	CCDC68
ENSG00000181472	ZBTB2
ENSG00000244513	AC109587.1
ENSG00000136247	ZDHHC4
ENSG00000186318	BACE1
ENSG00000145850	TIMD4
ENSG00000113161	HMGCR
ENSG00000106069	CHN2
ENSG00000152359	POC5
ENSG00000117899	MESD
ENSG00000177058	SLC38A9
ENSG00000094880	CDC23
ENSG00000187257	RSBN1L
ENSG00000142945	KIF2C
ENSG00000118197	DDX59
ENSG00000138434	SSFA2
ENSG00000213846	AC098614.1
ENSG00000197912	SPG7
ENSG00000174485	DENND4A
ENSG00000157933	SKI
ENSG00000186153	WWOX
ENSG00000141858	SAMD1
ENSG00000213949	ITGA1
ENSG00000111707	SUDS3
ENSG00000167842	MIS12
ENSG00000069431	ABCC9
ENSG00000138115	CYP2C8
ENSG00000132122	SPATA6
ENSG00000213551	DNAJC9
ENSG00000151135	TMEM263
ENSG00000136824	SMC2
ENSG00000108100	CCNY
ENSG00000248734	AC008906.1
ENSG00000132964	CDK8
ENSG00000135476	ESPL1
ENSG00000197969	VPS13A
ENSG00000185924	RTN4RL1
ENSG00000182481	KPNA2

ENSG00000113211	PCDHB6
ENSG00000143222	UFC1
ENSG00000133138	TBC1D8B
ENSG00000146066	HIGD2A
ENSG00000113811	SELENOK
ENSG00000008086	CDKL5
ENSG00000180900	SCRIB
ENSG00000198917	SPOUT1
ENSG00000164978	NUDT2
ENSG00000160606	TLCD1
ENSG00000170293	CMTM8
ENSG00000166803	PCLAF
ENSG00000086061	DNAJA1
ENSG00000119596	YLPM1
ENSG00000155363	MOV10
ENSG00000115255	REEP6
ENSG00000101751	POLI
ENSG00000116199	FAM20B
ENSG00000221988	PPT2
ENSG00000256690	AP001160.1
ENSG00000170667	RASA4B
ENSG00000246695	RASSF8-AS1
ENSG00000101365	IDH3B
ENSG00000156521	TYSND1
ENSG00000139133	ALG10
ENSG00000185658	BRWD1
ENSG00000238273	AC108058.1
ENSG00000169740	ZNF32
ENSG00000087460	GNAS
ENSG00000173848	NET1
ENSG00000099250	NRP1
ENSG00000254641	AC091564.3
ENSG00000239002	SCARNA10
ENSG00000135346	CGA
ENSG00000169562	GJB1
ENSG00000172478	C2orf54
ENSG00000169994	MYO7B
ENSG00000149452	SLC22A8
ENSG00000175899	A2M
ENSG00000163586	FABP1
ENSG00000115009	CCL20
ENSG00000077522	ACTN2
ENSG00000159625	DRC7
ENSG00000163501	IHH
ENSG00000100665	SERPINA4

ENSG00000082293	COL19A1
ENSG00000137731	FXYD2
ENSG00000203877	RIPPLY2
ENSG00000249201	CTD-3080P12.3
ENSG00000153822	KCNJ16
ENSG00000169245	CXCL10
ENSG00000142484	TM4SF5
ENSG00000079689	SCGN
ENSG00000072858	SIDT1
ENSG00000146151	HMGCLL1
ENSG00000139874	SSTR1
ENSG00000084674	APOB
ENSG00000145626	UGT3A1
ENSG00000276772	AC025271.4
ENSG00000196660	SLC30A10
ENSG00000233377	MTND4P20
ENSG00000178821	TMEM52
ENSG00000044012	GUCA2B
ENSG00000261586	AC068987.4
ENSG00000214491	SEC14L6
ENSG00000165300	SLITRK5
ENSG00000124440	HIF3A
ENSG00000184012	TMPRSS2
ENSG00000179603	GRM8
ENSG00000135447	PPP1R1A
ENSG00000095932	SMIM24
ENSG00000231439	WASIR2
ENSG00000180767	CHST13
ENSG00000232872	CTAGE3P
ENSG00000246877	DNM1P35
ENSG00000123405	NFE2
ENSG00000152377	SPOCK1
ENSG00000160593	JAML
ENSG00000152578	GRIA4
ENSG00000137033	IL33
ENSG00000104044	OCA2
ENSG00000151023	ENKUR
ENSG00000250919	UGT2B26P
ENSG00000184672	RALYL
ENSG00000226887	ERVMER34-1
ENSG00000120054	CPN1
ENSG00000054179	ENTPD2
ENSG00000274874	AC068790.8
ENSG00000261002	AC036103.1
ENSG00000258792	AL137230.1

ENSG00000234460	AL772337.3
ENSG00000250687	AC146944.2
ENSG00000233523	PHBP5
ENSG00000229666	MAST4-AS1
ENSG00000259420	AC046168.2
ENSG00000276744	AC105137.3
ENSG00000173338	KCNK7
ENSG00000123999	INHA
ENSG00000233588	CYP51A1P2
ENSG00000251204	AC027313.1
ENSG00000225493	LINC01107
ENSG00000188993	LRRC66
ENSG00000166006	KCNC2
ENSG00000081051	AFP
ENSG00000229314	ORM1
ENSG00000175707	KDF1
ENSG00000226774	AC073910.1
ENSG00000260892	AC105020.4
ENSG00000261056	AC079416.1
ENSG00000079557	AFM
ENSG00000119411	BSPRY
ENSG00000205045	SLFN12L
ENSG00000174498	IGDCC3
ENSG00000144820	ADGRG7
ENSG00000230795	HLA-K
ENSG00000161267	BDH1
ENSG00000251920	RNA5SP216
ENSG00000110195	FOLR1
ENSG00000255082	GRM5-AS1
ENSG00000163749	CCDC158
ENSG00000165140	FBP1
ENSG00000277653	CDKN2B-AS
ENSG00000181885	CLDN7
ENSG00000196440	ARMCX4
ENSG00000271916	AC012467.1
ENSG00000273353	AL008718.3
ENSG00000232412	AL121601.1
ENSG00000248367	AC008610.1
ENSG00000253618	GRPEL2-AS1
ENSG00000270587	AC046185.2
ENSG00000277463	AC080038.2
ENSG00000254731	AP003059.1
ENSG00000279845	AC097372.4
ENSG00000272432	AL031432.3
ENSG00000225726	AC007000.2

ENSG00000233942	AC004012.1
ENSG00000268836	Z69706.1
ENSG00000264475	AP005062.1
ENSG00000228204	AC004830.1
ENSG00000254740	AP003396.3
ENSG00000215241	LINC02449
ENSG00000215086	NPM1P24
ENSG00000262558	AC129507.3
ENSG00000249212	ATP1B1P1
ENSG00000278719	MCM8-AS1
ENSG00000231615	AL645568.2
ENSG00000237624	OXCT2P1
ENSG00000171989	LDHAL6B
ENSG00000124568	SLC17A1
ENSG00000268996	MAN1B1-AS1
ENSG00000258676	AC091544.2
ENSG00000176753	C15orf56
ENSG00000165972	CCDC38
ENSG00000284196	AL158064.2
ENSG00000278937	AL137141.1
ENSG00000198870	STKLD1
ENSG00000236283	AC019197.1
ENSG00000165828	PRAP1
ENSG00000174348	PODN
ENSG00000136960	ENPP2
ENSG00000162551	ALPL
ENSG00000235848	RMDN2-AS1
ENSG00000171724	VAT1L
ENSG00000111713	GYS2
ENSG00000250061	AC091976.1
ENSG00000228817	BACH1-IT2
ENSG00000147614	ATP6V0D2
ENSG00000259772	AC012236.1
ENSG00000229512	AC068580.1
ENSG00000254762	AP001107.7
ENSG00000130545	CRB3
ENSG00000225447	RPS15AP10
ENSG00000259786	LINC02109
ENSG00000184414	IRS3P
ENSG00000232810	TNF
ENSG00000183682	BMP8A
ENSG00000225988	LAMP5-AS1
ENSG00000260337	AC091544.5
ENSG00000272568	AC005162.3
ENSG00000167723	TRPV3

ENSG00000275234	AC010503.4
ENSG00000142149	HUNK
ENSG00000230445	LRRC37A6P
ENSG00000123453	SARDH
ENSG00000250421	AC106798.1
ENSG00000238184	CD81-AS1
ENSG00000260388	LINC00562
ENSG00000169594	BNC1
ENSG00000267795	SMIM22
ENSG00000227067	DPPA3P1
ENSG00000225206	MIR137HG
ENSG00000259203	AC016044.1
ENSG00000171643	S100Z
ENSG00000273312	AL121749.1
ENSG00000230043	TMSB4XP6
ENSG00000281566	AL157778.1
ENSG00000132000	PODNL1
ENSG00000220785	MTMR9LP
ENSG00000124564	SLC17A3
ENSG00000268112	AC008761.1
ENSG00000280110	AC087893.2
ENSG00000214578	HMGN2P15
ENSG00000239884	RN7SL608P
ENSG00000241120	HMGN1P8
ENSG00000246225	AC006299.1
ENSG00000232059	AL451007.1
ENSG00000176268	CYCSP34
ENSG00000251050	AC112184.1
ENSG00000272043	AC016405.2
ENSG00000237343	AC246785.3
ENSG00000266538	AC005838.2
ENSG00000201581	RN7SKP78
ENSG00000254842	LINC02551
ENSG00000276298	AC234782.5
ENSG00000276505	AP000892.2
ENSG00000254138	AC113386.1
ENSG00000278017	AC064801.1
ENSG00000204904	LINC01545
ENSG00000271874	AC025754.2
ENSG00000256361	AC027544.1
ENSG00000259521	INO80-AS1
ENSG00000271218	AL033384.2
ENSG00000279130	AC091925.1
ENSG00000253194	AL365275.1
ENSG00000262402	AC090617.3

ENSG00000271424	AL157791.2
ENSG00000230769	Z98048.1
ENSG00000249426	AC093206.1
ENSG00000249092	AC008945.1
ENSG00000233956	BTF3P6
ENSG00000258857	AL359397.1
ENSG00000238251	AL133477.1
ENSG00000229531	Z99127.1
ENSG00000270679	AC138473.1
ENSG00000251393	AC005280.1
ENSG00000272459	AC139795.3
ENSG00000249996	AC106786.2
ENSG00000158869	FCER1G
ENSG00000224451	ATP5F1P1
ENSG00000249412	AC010285.2
ENSG00000275784	AL034549.1
ENSG00000256615	AC010197.1
ENSG00000259364	AC013356.3
ENSG00000254542	NAV2-AS3
ENSG00000225328	LINC01594
ENSG00000280511	AL591030.1
ENSG00000256588	AC027544.2
ENSG00000259359	AC012409.1
ENSG00000243250	AP002884.1
ENSG00000274598	AC087893.1
ENSG00000276166	AC092118.2
ENSG00000228097	MTATP6P11
ENSG00000229155	LINC02038
ENSG00000203740	METTL11B
ENSG00000223779	AC239800.1
ENSG00000260088	AL445483.1

Table S3: Summary of experimental results from cells transduced with a single gene or all nine (9g-Li-7) genes

Tumorigenicity in mice, spheroid formation assay, chemosensitivity assay, CD13/CD166 expression, and cell proliferation assay were compared using cells transduced with a single gene or 9g-Li-7.

n.s., no significance; ↓ decreased growth rate; ↑increased growth rate.

Gene name	Tumorigenicity in mice	Number of spheres		5-FU resistance	CD13/166 expression	Cell proliferation
		>100μm	>200μm			
ENPP2	n.s.	n.s.	n.s.	n.s.	n.s.	↓
SCGN	n.s.	n.s.	n.s.	n.s.	n.s.	↑
FGFR4	n.s.	n.s.	n.s.	n.s.	n.s.	↓
MCOLN3	n.s.	n.s.	n.s.	n.s.	n.s.	↓
KCNJ16	n.s.	n.s.	n.s.	n.s.	n.s.	n.s.
SMIM22	n.s.	n.s.	n.s.	n.s.	n.s.	n.s.
SMIM24	n.s.	n.s.	n.s.	n.s.	n.s.	n.s.
SERPINH1	n.s.	n.s.	n.s.	n.s.	n.s.	n.s.
TMPRSS2	n.s.	n.s.	n.s.	n.s.	n.s.	↑
9 genes	n.s.	n.s.	n.s.	n.s.	n.s.	↓
